# Expression divergence of expansin genes drive the heteroblasty in *Ceratopteris chingii*

**DOI:** 10.1186/s12915-023-01743-7

**Published:** 2023-11-06

**Authors:** Yue Zhang, Yves Van de Peer, Bei Lu, Sisi Zhang, Jingru Che, Jinming Chen, Kathleen Marchal, Xingyu Yang

**Affiliations:** 1 Aquatic Plant Research Center, Wuhan Botanical Garden, Chinese Academy of Sciences, Wuhan, 430074 China; 2 Department of Plant Biotechnology and Bioinformatics, Ghent University, 9052 Ghent, Belgium; 3 VIB Center for Plant Systems Biology, 9052 Ghent, Belgium; 4 Department of Biochemistry, Genetics and Microbiology, University of Pretoria, Pretoria, 0028 South Africa; 5 College of Horticulture, Academy for Advanced Interdisciplinary Studies, Nanjing Agricultural University, Nanjing, 210095 China; 6 University of Chinese Academy of Sciences, Beijing, 100049 China; 7 Wuhan Institute of Landscape Architecture, Wuhan, 430081 China; 8 Department of Information Technology, IDLab, IMEC, Ghent University, 9052 Ghent, Belgium; 9 Hubei Ecology Polytechnic College, Wuhan, 430200 China

**Keywords:** Expansin, Ferns, *Ceratopteris chingii*, Phylogeny, Coexpression network, Heteroblasty

## Abstract

**Background:**

Sterile-fertile heteroblasty is a common phenomenon observed in ferns, where the leaf shape of a fern sporophyll, responsible for sporangium production, differs from that of a regular trophophyll. However, due to the large size and complexity of most fern genomes, the molecular mechanisms that regulate the formation of these functionally different heteroblasty have remained elusive. To shed light on these mechanisms, we generated a full-length transcriptome of *Ceratopteris chingii* with PacBio Iso-Seq from five tissue samples. By integrating Illumina-based sequencing short reads, we identified the genes exhibiting the most significant differential expression between sporophylls and trophophylls.

**Results:**

The long reads were assembled, resulting in a total of 24,024 gene models. The differential expressed genes between heteroblasty primarily involved reproduction and cell wall composition, with a particular focus on expansin genes. Reconstructing the phylogeny of expansin genes across 19 plant species, ranging from green algae to seed plants, we identified four ortholog groups for expansins. The observed high expression of expansin genes in the young sporophylls of *C. chingii* emphasizes their role in the development of heteroblastic leaves. Through gene coexpression analysis, we identified highly divergent expressions of expansin genes both within and between species.

**Conclusions:**

The specific regulatory interactions and accompanying expression patterns of expansin genes are associated with variations in leaf shapes between sporophylls and trophophylls.

**Supplementary Information:**

The online version contains supplementary material available at 10.1186/s12915-023-01743-7.

## Background

Heteroblasty is a developmental trajectory event where plants have rapid ontogenetic changes across multiple traits, as exemplified by plants switching from distinct juvenile to adult leaves [1]. Heteroblastic leaves, which exhibit different morphologies and functions, enable plants to adapt to environmental heterogeneity, serving as a prime example of adaptive evolution. For thriving in undulating air-water environments, amphibious and aquatic plants tend to form heteroblastic leaves at different developmental stages [2, 3]. While previous studies uncovered some of the underlying differences of heteroblasty at the morphological, physiological, and molecular levels in aquatic seed plants [4–7], the mechanism behind sterile-fertile leaf dimorphy, i.e., heteroblasty with divergent functionalities, remains relatively unexplored in aquatic ferns.

**Fig. 1:**
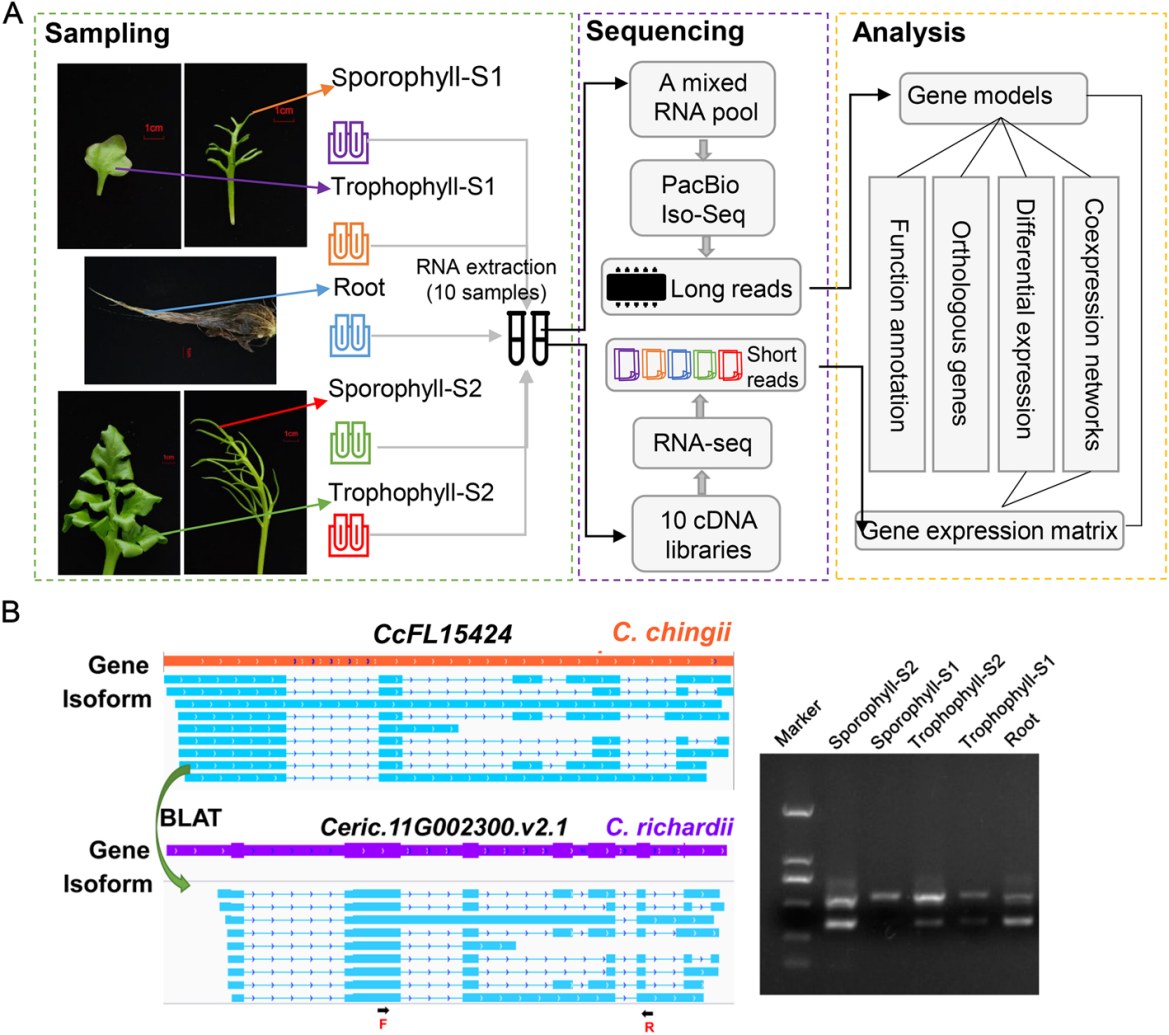
Generation of the dataset and gene models for *C. chingii*. **A** Flowchart for gene annotation by PacBio full-length sequencing and Illumina RNA-seq for *C. chingii*. Sampling: photos show 5 tissue samples from different development stages (two biological repeats per developmental stage), of which the root system was shown but the single root sample was collected, and the total RNA of 5 tissue samples was extracted. Sequencing: RNA of each sample was used to build a cDNA library. Each library was subjected to Illumina short-read sequencing. In parallel, an RNA pool was made by mixing equal quantities of RNAs taken from each 10 samples. This pool was subjected to PacBio full-length sequencing. Analysis: reliable gene models of *C. chingii* were assembled by combining long with short read sequence data. In addition, mapping the short reads on the inferred gene models allowed quantifying the expression of each gene per developmental stage (expression matrix). **B** RT-PCR validation of a high-confident gene model. The isoforms of *CpFL15424* were mapped to *C. richardii* using BLAT and further clustered in *Ceric.11G002300.v2.1* gene regions. The arrows show the loci of the PCR primers (F, forward, and R, reverse) on the last isoform. The RT-PCR amplifications in *C. chingii* for the different tissues are shown in the right panel

Ferns are an ancient lineage of vascular plants and occupy a key phylogenetic position as a sister to the seed plants [8–10]. Given their wide distribution and diverse habitats, ranging from submerged environments to alpine environments, ferns exhibit a remarkable diversity in leaf shape, and sterile-fertile heteroblasty is common [11, 12]. The genus *Ceratopteris*, a model aquatic fern with heteroblastic leaves, displays distinctive patterns of genomic evolution compared to terrestrial ferns and seed plants [13, 14]. Among the species in this genus, *C. chingii* (2*n* = 78) is a leptosporangiate fern species closely related to the model system *C. richardii* (2*n* = 78), and it has a wide geographic distribution in Asia [15–18]. In *C. chingii*, the abaxial leaf surface of the sporophyll is curved to enclose the simple-structured sporangium originating from a single superficial initial cell in the leaf through transverse division [19, 20]. On the other hand, the trophophyll, which is responsible for primary buoyancy and plant floating, grows with an unfolded leaf surface (Fig. 1A) [15, 21]. The dramatic morphological differences between sporo- and trophophylls make *C. chingii* an ideal material for studying the molecular mechanism underlying heteroblastic leaf formation in aquatic ferns. Additionally, expansin (EXP), a plant cell-wall loosening protein, played a crucial role in cell shape plasticity and leaf morphology by facilitating the disassembly, remodeling, and adjustment of the cell wall throughout leaf development [22–24]. However, it remains largely unknown whether the expression patterns of expansins are distinct in heteroblastic leaves, particularly in ferns.

Because the heteroblastic leaves were produced by the same plant, differences in the gene expression and regulation play important roles [25, 26]. However, due to the large genome size of most ferns, reference assembly by whole genome sequencing is not straightforward and limits genomic research [27]. Recently, only a few fern genomes have been sequenced [15, 28–30], including five homosporous ferns: *C. richardii* [15], *Adiantum capillus-veneris* [30], *A. nelumboides* [31], *Marsilea vestita* [32], and *Alsophila spinulosa* [28], as well as two heterosporous ferns: *Azolla filliculoides* and *Salvinia cucullata* [29]. *C. chingii* belongs to the homosporous ferns, which generally have relatively large genomes compared to their heterosporous counterparts [18, 27, 33]. Full-length transcriptome sequencing has provided an attractive alternative for gathering information on gene transcripts and predicting gene models without a reference genome [34, 35]. The strategy of combining long- and short-read transcriptome sequencing has been widely applied to study species-specific traits in seed plants [36, 37].

In this study, we applied the same strategy of combining long- and short-read RNA sequencing to construct reference gene models and study gene expression relevant to sporophyll, trophophyll, and root tissues in *C. chingii*. Given the key roles of expansin genes in the leaf development of seed pants [22, 38], we aimed to investigate the expression patterns of *C. chingii* expansin (*CcEXP* genes) and how they regulated the formation of heteroblastic leaves in *C. chingii*. We found that *CcEXP* genes were highly expressed in young sporophylls and trophophylls. Building on the previous study of expansin in several ferns [39, 40], we further constructed phylogenies of the expansin gene family and identified, by means of coexpression analysis, regulatory factors that positively or negatively affect *CcEXP* gene expression. In addition, we compared the expression conservation of orthologs between ferns and lycophytes, as well as the co-expressed orthologous relationships between *C. chingii* and the model seed plant *Arabidopsis thaliana*.

## Results

### Gene annotation by full-length transcriptome sequencing

To obtain a representative transcriptome for *C. chingii*, we performed a PacBio full-length RNA sequencing (Fig. 1A) and obtained a total of 915,702 reads with an average length of 89,338 bp (Additional file 1: Fig. S1, Table 1). After the removal of low-quality reads, circular consensus sequencing reads were classified into 513,861 (73.49%) full-length non-chimeric reads (FLNCs) and 155,285 (26.51%) non-full-length reads (Table 1). By error-correcting these FLNCs using non-full-length reads and Illumina Seq short reads, 37,428 non-redundant high-quality full-length transcripts with an average length of 1373 bp and an N50 of 1563 bp were obtained (Additional file 1: Fig. S1, Table 1). Using these non-redundant high-quality full-length transcripts, we constructed de novo gene models. In total, we obtained 24,024 transcript families, each of which corresponded to one gene model. Among these transcript families, 9267 were UniTransModels and 14,757 transcripts belonged to singleton genes with one isoform only. BUSCO analysis of the gene models showed that our gene models achieved 72.2% completeness according to the eukaryote (odb10) core gene dataset, indicating a comprehensive transcriptome dataset.

**Table 1:** Summary of the full-length transcript sequencing of *C. chingii* on the PacBio Sequal platform

Category	Nanopore full-length sequencing
Raw read number	915,702
Base number of raw reads (Gbp)	81.81
Mean length of raw reads (bp)	89,338
Circular consensus sequencing reads number	669,146
FLNC number	513,861
FLNC percentage (%)	73.49%
N50 of FLNC (bp)	1551
Mean length of FLNC (bp)	1368
Max length of FLNC (bp)	3181
Non-full-length number	155,285
Non-full-length percentage (%)	26.51%

Full-length transcriptome sequencing only captures information on exonic regions. To predict the intronic regions of the gene models as well, the high-quality full-length transcripts were mapped to the chromosome-level reference assembly of *C. richardii*, assuming a high similarity in gene content and structure between *C. richardii* and *C. chingii*. If all transcripts were assigned to a *C. chingii* gene model mapped to a unique gene locus on the *C. richardii* genome, the gene model was regarded as a high-confident homologous match. This was the case for 18,079 gene models (75.25%), and the sequence intron region was transferred from the *C. richardii* to *C. chingii* gene. *C. chingii* gene models with ambiguous mapping (4262 (17.74%)) or those without mapping (1692 (7.04%)) were classified as low-confident gene models. To validate the accuracy of the inferred gene structure of the *C. chingii* genes, 10 randomly selected splicing events identified in the inferred gene models were subjected to RT-PCR (Fig. 1B, Additional file 1: Fig. S2). The size of all of the amplified products corresponded to the size of the exons based on the predicted gene structure. This indicates a high similarity between the gene coding regions of both fern genomes exists (i.e., high-confident genes in *C. chingii*), which further supports the validity in extrapolation of the gene structure information.

To functionally annotate our gene models in *C. chingii*, we conducted a comprehensive local alignment against various sequence databases, including GO, KEGG, KOG, NCBI Nr, and SwissProt. A total of 19,565 (81.43%) gene models showed matches to sequences with known annotation in at least one of the five databases. Furthermore, 5079 (21.14%) gene models exhibited matches with annotation in all five databases (Additional file 1: Fig. S3). To annotate transcription factors [41, 42], the *C. chingii* gene models were mapped to the PlantTFDB database. This analysis led to the identification of 603 transcription factors belonging to 72 families, with the C3H and the bHLH families being the largest (Additional file 1: Fig. S4). Next to protein-coding genes, we also discovered 303 long non-coding RNAs (LncRNAs), potentially involved in the post-transcriptional regulation.

### Expansin (EXP) genes are differentially expressed between trophophylls and sporophylls

**Fig. 2:**
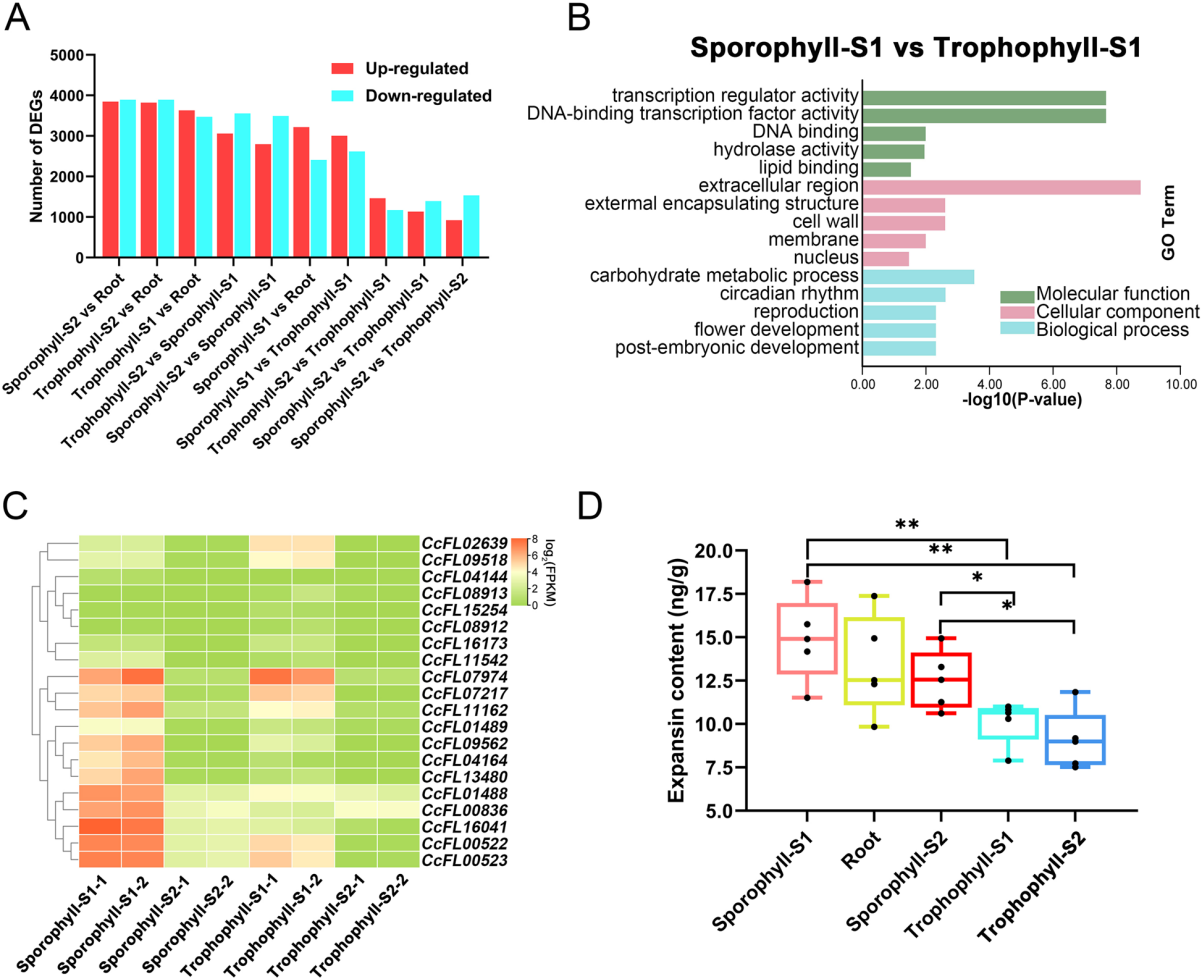
Differential gene expression patterns in *C. chingii*. **A** Bar chart showing the number of differentially expressed genes between five tissues. Red bars indicate upregulated genes, and blue bars are downregulated genes. **B** GO enrichment of the genes that are differentially expressed between young sporophylls and young trophophylls. **C** Heatmap showing the expression pattern of 20 expansin genes in eight tissue samples. **D** Box graph showing the expansin content in five tissues. The significance of the differences in the expansin content between tissues was tested by the *t*-test. *p-value < 0.05, **p-value < 0.01

To quantify the expression level of genes in different tissues of *C. chingii*, we conducted short-read RNA-seq on five tissue samples, with two biological repeats per tissue sample (non-pooled). The resulting short reads were mapped to the identified gene models to determine the read counts and gene expression levels, measured as FPKM. To identify tissue-dependent expression patterns that contribute to tissue morphology [43], we performed differential expression analysis between any two pairs of tissues (Fig. 2A). The largest number of differentially expressed genes (DEGs) was identified between trophophyll-S2 and root (7741), with 3846 upregulated genes and 3895 downregulated genes (Fig. 2A). Conversely, the smallest number of DEGs were observed between sporophyll-S2 and trophophyll-S2, with 2461 genes, of which 925 upregulated and 1536 downregulated in the sporophyll compared to the trophophyll (Fig. 2A).

We found three significantly enriched GO terms “reproduction,” “flower development,” and “post-embryonic development,” in the comparison of sporophyll-S1 and trophophyll-S1, suggesting their potential involvement in sexual reproduction. Additionally, three significantly enriched GO terms in the cellular component class were found overrepresented between trophophyll and sporophyll, namely “extracellular region,” “external encapsulating structure,” and “cell wall” (Fig. 2B, Additional file 1: Fig. S5), all of which are associated with cell wall morphology. Furthermore, based on their functional annotation of the Swissprot database, 20 genes within these three significantly enriched GO terms were predicted to be expansin (EXP) proteins and were the major component. These EXP genes exhibited differential expression in at least one of the four comparisons between sporophyll and trophophyll (Additional file 1: Fig. S6), i.e., trophophyll-S1 vs sporophyll-S1, trophophyll-S1 vs sporophyll-S2, trophophyll-S2 vs sporophyll-S1, and trophophyll-S2 vs sporophyll-S2. We visualized their expression pattern in a heatmap (Fig. 2C), which revealed that 12 (60%) of the EXP genes show higher expression levels in sporophyll-S1 compared to other tissue samples (Fig. 2C). This expression pattern suggests that the increased expression of EXP gene in the sporophyll-S1 may influence cell morphology and potentially contribute to sporophyll formation.

To validate the upregulation of EXP genes at the protein levels, we quantified the EXP content in the five same tissue samples used for RNA-seq. Notably, the EXP content was found to be the highest in sporophyll-S1 and showed significant differences when compared to trophophyll-S1 and trophophyll-S2 (*t*-test, *p* < 0.01, Fig. 2D). Similarly, significantly more expansin protein was identified in sporophyll-S2 than in trophophyll-S1 and trophophyll-S2 (*t*-test, *p* < 0.05, Fig. 2D). These findings suggest that the increased abundance of expansin proteins may contribute to the more intricate cellular organization and distinct tissue phenotype observed in sporophyll compared to trophophyll. Overall, this highlights the significant association between differentially expressed EXP genes and the divergent phenotypes of heteroblastic leaves in *C. chingii*.

### Identification and phylogenetic analysis of expansin gene family in C. chingii

**Fig. 3:**
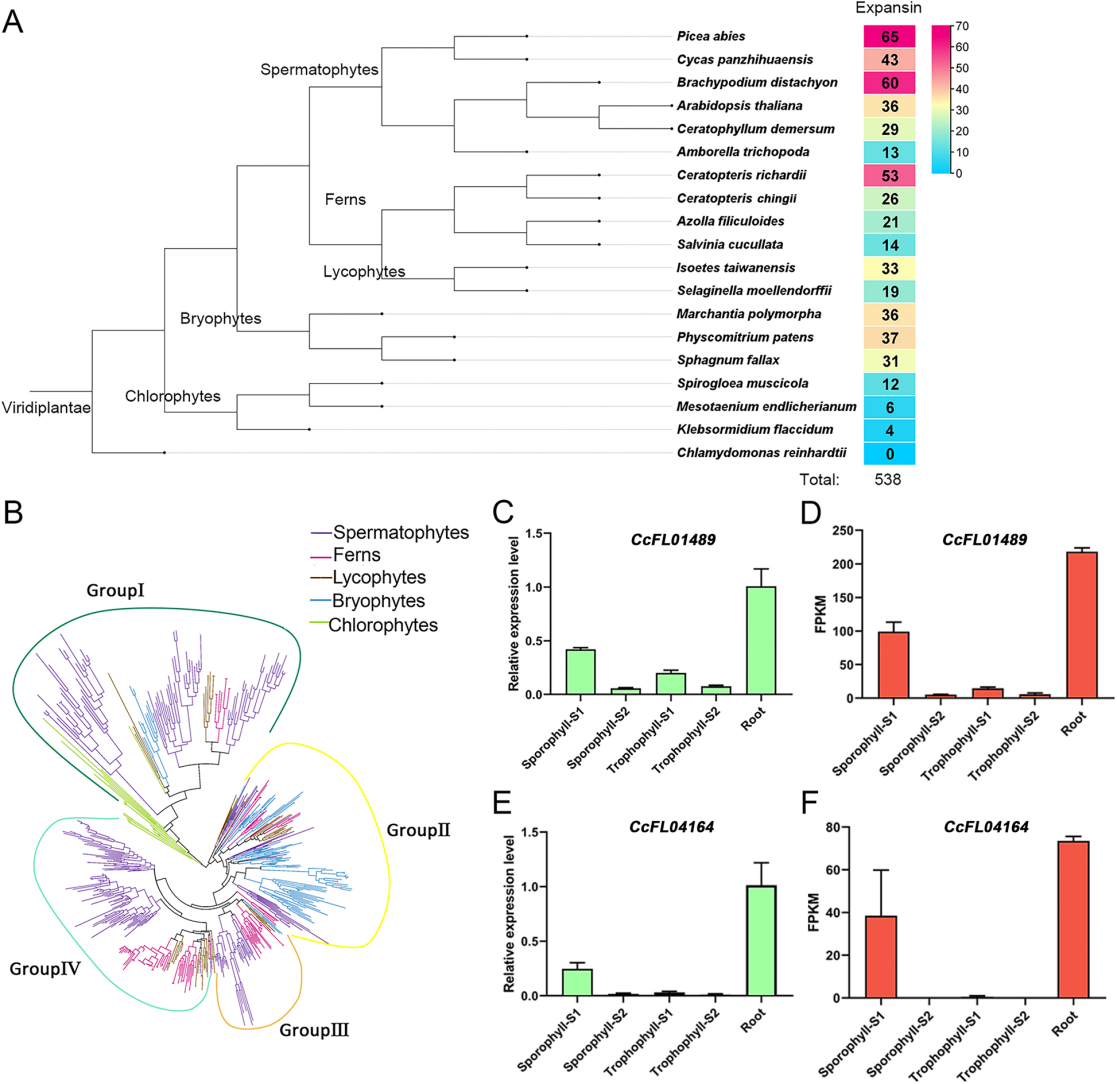
Phylogeny of the expansin gene family. **A** Evolutionary tree topology representing the species from which EXP genes were retrieved. Numbers indicate the number of identified EXP sequences in these 19 species. **B** Phylogenetic tree of expansins, built with maximum likelihood. Branches are colored, depending on the species of origin from which the extant sequence was derived (spermatophyte, ferns, lycophytes, bryophyte, and chlorophyte). Four different phylogenetic groups of EXP genes can be distinguished. **C**–**F** Bar plots showing the qRT-PCR results and RNA-seq derived expression levels of *CcFL01489* and *CcFL04164* in five tissues

Previous phylogenetic analyses have classified expansins into at least four subfamilies [44, 45]. However, these analyses were restricted to seed plants and a few ferns [39, 40]. To expand the phylogeny to include more fern lineages, we first identified expansin genes in *C. chingii*. We selected all expansin-like genes that contained the conserved DPBB-1 and the Expansin_C domain, known to be a characteristic of the expansin protein function. This resulted in 26 *CcEXPs* from the 28 candidate genes, with an average of 271 amino acids (Additional file 2: Table S1). We applied similar criteria to identify candidate expansin genes in 19 additional plant genomes, including an outgroup (*Chlamydomonas reinhardtii*), chlorophytes (*Klebsormidium flaccidum*, *Mesotaenium endlicherianum*, *Spirogloea muscicola*), bryophytes (*Marchantia polymorpha*, *Physcomitrium patens*, *Sphagnum fallax*), lycophytes (*Isoetes taiwanensis, Selaginella moellendorffii*), ferns (*Azolla filiculoides*, *C. chingii*, *C. richardii*, *Salvinia cucullata*), and spermatophytes (*Amborella trichopoda*, *Arabidopsis thaliana*, *Brachypodium distachyon*, *Ceratophyllum demersum*, *Cycas panzhihuaensis*, *Picea abies*). This resulted in 538 EXP candidate genes (Fig. 3A). By including more genomes, the number of EXP genes largely increased (Fig. 3A). Interestingly, the basal species within each cluster have relatively fewer EXP genes compared to other species in the same cluster (Fig. 3A). Using the maximum likelihood (ML) method, we generated a phylogeny of all EXP genes, with Chlorophyta as an early divergent lineage (Fig. 3B). In seed plants, the expansins are subdivided into 4 subfamilies: EXPA, EXPB, EXLA, and EXLB. *C. chingii* only has representatives in the EXPA and EXPB subfamilies (Additional file 1: Fig. S7, Additional file 2: Table S1). We also observed the absence of EXLA and EXLB genes also for other ferns, suggesting that ferns lack these subfamily expansin genes. Using MEME, we identified ten conserved motifs in the *C. chingii* EXP proteins (Additional file 1: Fig. S8).

The phylogenetic tree of EXP genes is composed of four distinct phylogenetic groups, labeled as group I to group IV (Fig. 3B, Additional file 2: Table S2). Group I consisted of EXPB, EXLA, and EXLB genes, while groups II~IV only contained EXPA genes (Fig. 3B). Group I EXP genes are found in all 18 plant genomes, whereas group II and group III consist of EXP genes from 15 plant genomes, excluding chlorophytes (Additional file 1: Fig. S9). Interestingly, group IV exclusively contains EXP genes from vascular plants, including lycophytes, ferns, and spermatophytes. These findings suggest that vascular plants possess a class of specific EXP genes, and their diversification likely contributes to the development of the vascular system.

In *C. chingii*, 69.23% of *CcEXP* genes (18 genes) were present in group III and group IV, with 14 *CcEXP* genes showing differentially expressed in at least one of the four comparisons mentioned above (Additional file 1: Fig. S6). Our results indicate that seven and five *CcEXP* genes are relatively more highly expressed in sporophyll-S1 and trophophyll-S1, respectively, compared to other leaf tissue. These results highlight the distinct expression patterns of *CcEXP* genes in these two groups. To validate the accuracy of the expression pattern of these *CcEXP* genes, we performed qRT-PCR experiments in all five tissues (Additional file 1: Fig. S10 and Fig. 3C–F for the tissue-specific *CcEXP* genes). Due to the high sequence similarity between gene pairs *CcFL01488*-*CcFL01489* and *CcFL08912*-*CcFL08913*, we tested only one gene from each pair, namely *CcFL01489* and *CcFL08913* (Fig. 3C and Additional file 1: Fig. S10I). The relative expression level of the tested *CcEXP* genes in the five tissues showed an average correlation of 0.84 with their FPKM expression level (Additional file 2: Table S3), confirming the expression patterns derived from RNA-seq data.

### Gene co-expression networks of CcEXP genes representative of different phylogenetic groups

In line with previous studies [42], we employed coexpression analysis using the *CcEXP* genes as query genes to identify additional genes in *C. chingii* that are involved in the same process as those query genes and to infer transcription factors that regulate the *CcEXP* query genes. A total of 23 filtered *CcEXP* genes were utilized to construct a co-expression network, comprising two *CcEXP* genes from group I, five from group II, seven from group III, and nine from group IV. The degree of coexpression between a *CcEXP* query gene and other genes was evaluated using the Spearman correlation coefficient (*r*). Only a few strong coexpression relations (|*r*| > 0.95) were detected between the *CcEXP* query genes themselves, particularly within genes of the same group, indicating distinct expression behavior among different *CcEXP* genes. By applying a stringent threshold for correlation (|*r*| > 0.95 and *p*-value < 1e−5), 1501 genes were identified to be significantly positively (*r* > 0.95) or negatively (*r* < − 0.95) coexpressed with *CcEXP* genes, including 74 transcription factors and 26 lncRNAs. The majority of these genes (907, 60.42%) showed significantly coexpressed with one *CcEXP* gene only, while 594 (39.57%) genes showed a coexpression behavior with multiple *CcEXP* genes. Most of the significant coexpression relations (2511, 86.82%) identified using the Spearman correlation were ranked in the top 1%, confirming the robustness of the results. The limited overlap in coexpression relationships among *CcEXP* genes suggests their involvement in distinct regulatory networks and their different roles in tissue morphogenesis. Furthermore, no DEGs related to reproduction were identified as significant coexpressed genes with any *CcEXP* genes, indicating a rare interaction between spore development and heteroblastic leaf formation in *C. chingii*.

**Fig. 4:**
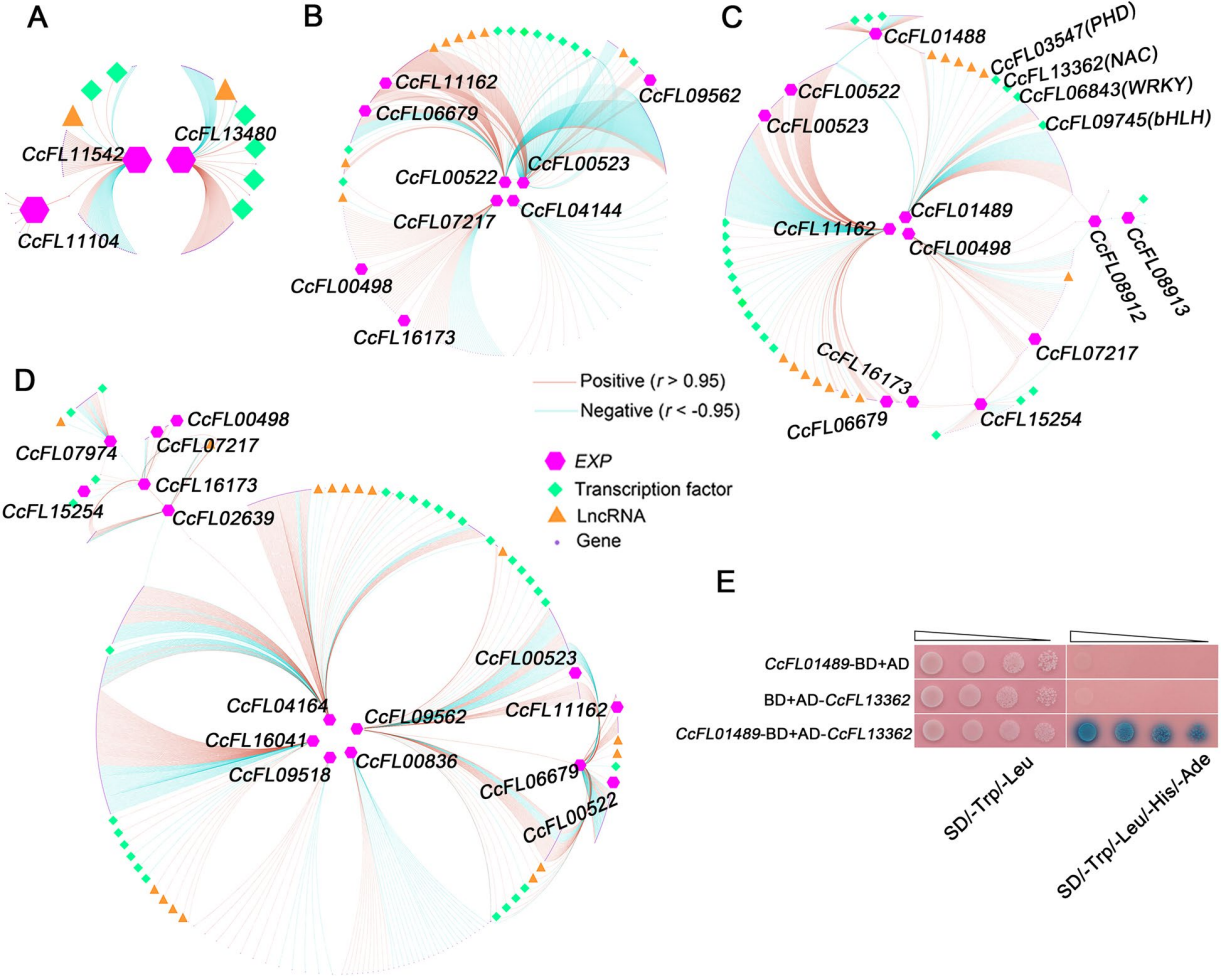
Coexpression interactions of *C. chingii* expansin genes. **A**–**D** Gene coexpression networks of the *CcEXP* genes belonging to the different phylogenetic groups: group I (**A**), group II (**B**), group III (**C**), group IV (**D**). The hexagons represent the *CcEXP* genes of a group, rhombus represent the transcription factors, triangles represent the lncRNA, and circles represent the genes coexpressed with the *CcEXP* genes. Red links represent significant (*r* > 0.95) positive coexpression interactions and blue links represent significant (*r* < − 0.95) negative coexpression interactions. **E** Yeast two-hybrid assay showing how *CcFL01489* interacts with NAC transcription factor (*CcFL13362*). Empty vector pGKBT7 (BD) and pGADT7 (AD) as the negative control. Recombinant plasmids were transformed into Y2H gold yeast strain and grown on selection medium at 30 °C for 3 days

Figure 4A–D depicts the specific coexpression network for each group of *CcEXP* genes. Each network was constructed using only the *CcEXP* genes from a particular group to calculate coexpression. In the coexpression networks of the group I and group II *CcEXP* genes, with the exception of one pair (*CcFL00522* and *CcFL00523*), most query *CcEXP* genes do not share any coexpressed genes (Fig. 4A, B). This suggests that *CcEXP* genes, which have a common ancestor in spermatophytes and bryophytes, have likely diverged in function throughout the species’ evolution. In contrast, the coexpression network of the group III and group IV *CcEXP* genes show more shared coexpressed genes, indicating a more similar level of expression regulation (Fig. 4C, D). GO enrichment analysis of the coexpressed genes reveals their involvement in the extracellular region and cell wall for group III and group IV *CcEXP* genes (Additional file 1: Fig. S11).

Given the significance of transcriptional regulation, we focused on identifying transcription factors and lncRNAs in each of the coexpression networks corresponding to the different phylogenetic groups. In these coexpression networks, members of the bHLH family were most abundant, followed by members of the WRKY family (Additional file 1: Fig. S12). Additionally, 37 lncRNA were predicted to interact with 17 *CcEXP* genes (Additional file 2: Table S4). Among *CcEXP* genes, 17 (73.91%) were predicted to be regulated by at least one transcription factor or one lncRNA. However, these inferred regulatory relationships were rarely shared between different *CcEXP* genes. Only the *CcEXP* genes of group III were regulated by at least one transcription factor or lncRNA. This suggests that for most *CcEXP* genes the regulatory interactions are quite specific.

Predicting regulatory relations based on coexpression is prone to false positives. To further validate the inferred regulatory interactions between *CcEXP* genes and transcription factors, the *CcFL01489* gene of group III along with four transcription factors, inferred to regulate this gene were subjected to a yeast two-hybrid assay (Fig. 4E, Additional file 1: Fig. S13). Since the *CcFL01489* gene exhibited a high expression level in sporophyll-S1 and the highest correlation coefficient between RNA-seq and RT-PCR (Additional file 2: Table S3), it might be associated with the formation of heteroblasty in *C. chingii*. We intentionally measured direct protein-protein interactions, rather than protein DNA interactions, as we did not have access to the non-coding sequence of the *CcEXP* genes in *C. chingii*. As shown in Fig. 4E, the positive transformants of the yeast strain (white) grow normally on the SD/-Trp/-Leu dropout medium. However, only one yeast strain (blue) harboring G1-BD+*CcFL13362* survived on the SD/-Trp/-Leu/-His/-Ade + X-α-gal dropout medium. Therefore, our results indicate that *CcFL01489* interacts with one transcription factor *CcFL13362* and that this interaction likely results in the observed high expression of *CcFL01489* in *C. chingii* sporophyll-S1.

### Expression patterns and coexpression interactions of EXP genes are largely species-specific

**Fig. 5:**
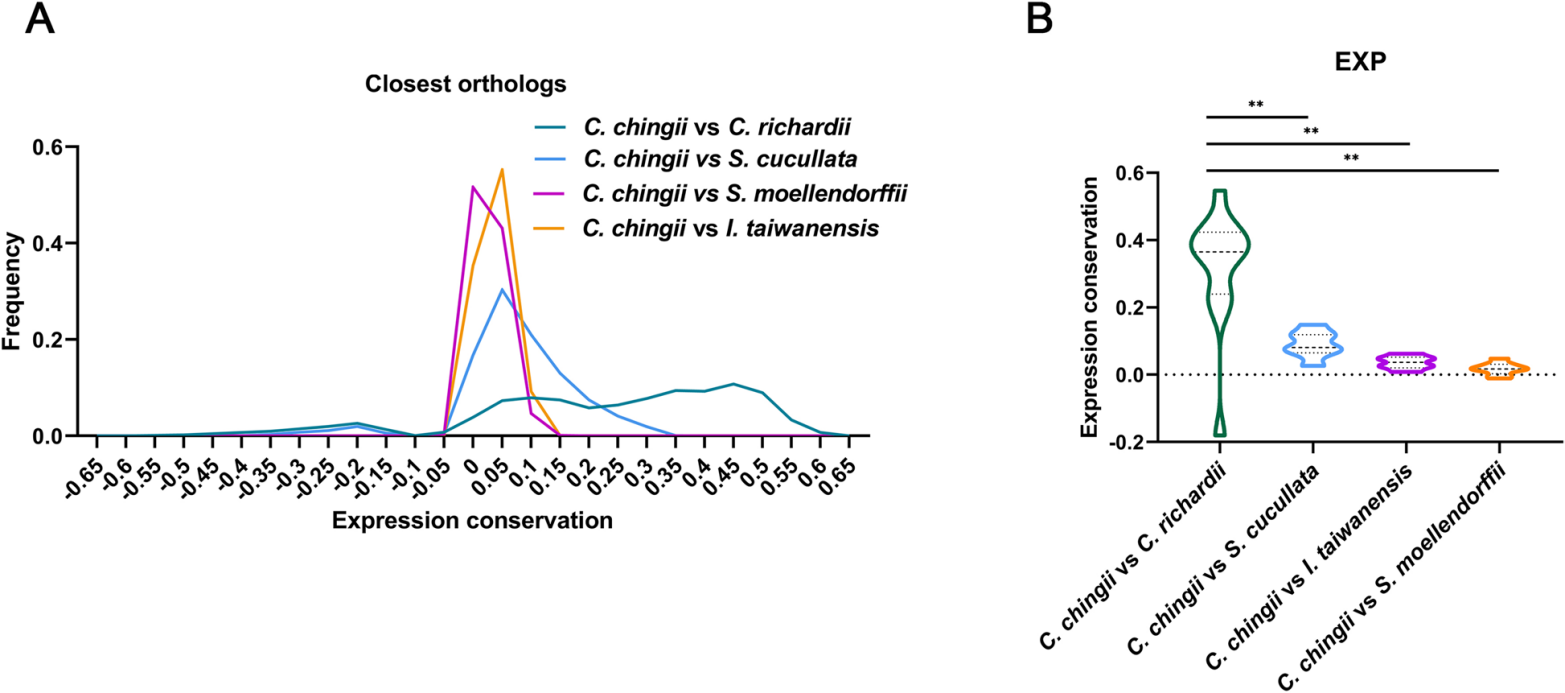
Expression conservation of orthologous gene pairs. These plots show the degree to which highly similar orthologs tend to have the same expression behavior in two compared species (expression conservation). Expression conservation (EC) and highly similar orthologs are defined as described in materials and methods. **A** The frequency plot shows the distribution of the EC values for the comparison of all highly similar orthologs between the two indicated species. **B** Violin plot showing the distribution of the EC values for the comparison of orthologous EXP gene pairs between the two indicated species. Significance in EC was assessed by the Mann-Whitney *U* test. *p-value < 0.05, **p-value < 0.01

As differences in the expression patterns of orthologs often relate to phenotypic interspecies differences [46], we investigated how EXP orthologs from different fern species differ in their expression behavior. To do this, we built the gene expression matrices of leaf and root samples (Additional file 2: Table S5) from three fern species (*C. chingii*, *C. richardii*, *S. cucullate*) and two lycophyte species (*I. taiwanensis*, *S. moellendorffii*) (see the “Methods” section). To account for the differences in the number and developmental stage of the tissue samples available for each species, we used the method described by Tirosh et al. [47] to determine the degree to which the expression behavior is conserved between pairs of highly similar orthologs in *C. chingii* and respectively each of the abovementioned species (EC expression conservation, see the “Materials” section). In our genome-wide analysis, considering all identified highly similar orthologous gene pairs in each species combination, we found the highest level of expression conservation (EC) for orthologous pairs of the closely related lineages *C. chingii* and *C. richardii* (Fig. 5A). However, when comparing *C. chingii* with other species (*S. cucullate*, *I. taiwanensis*, and *S. moellendorffii*), orthologous pairs showed, on average, a low level of EC (− 0.05 ~ 0.1), indicating a high divergence in expression behavior in leaf and root tissues (Fig. 5A) between ferns and between fern and lycophyte.

When we specifically analyzed the EC for orthologous pairs of EXP genes, we found that, consistent with genome-wide analysis, the EC level of the close orthologous EXP gene pairs was significantly higher in the comparison of *C. chingii vs C. richardii* than in comparisons of *C. chingii* with other ferns (Mann-Whitney *U* test, *p* < 0.05, Fig. 5B). Rarely, we observed the EC of close orthologous EXP genes in the top 5% EC values observed in the genome-wide comparison. This was true for each of the different species comparisons, further confirming that orthologous EXP genes evolved largely distinct expression patterns.

To investigate how the coexpression interactions of EXP genes belonging to a specific group evolved during species differentiation, we compared the gene coexpression network constructed for *C. chingii* involving the EXP genes in each phylogenetic group with that of the corresponding orthologous EXP genes in *C. richardii* and the outgroup *A. thaliana* (see the “Methods” section) (Additional file 1: Fig. S14). Similar to what we observed for *C. chingii*, coexpression relationships between EXP in *C. richardii* or *A. thaliana* were rarely significant (Additional file 1: Fig. S14), indicating the dramatically distinct expression patterns for this gene family, even within species. Moreover, the coexpression networks of EXP genes belonging to the same group in the comparisons between *C. richardii* and *C. chingii* and between *A. thaliana* and *C. chingii* contain transcription factors of the same family. For example, the MYB transcription factors were coexpressed with group III EXP genes in respective *C. chingii* and *C. richardii* coexpression networks; bHLH and bZIP transcription factors were coexpressed with group II EXP genes in respective *C. chingii* and *A. thaliana* coexpression networks. This indicates a conserved regulation of EXP genes and transcription factors between plant species. Genes coexpressed with the *AtEXP* genes of group IV (Additional file 1: Fig. S15A) were enriched for pathways involved in response to light and abiotic stimuli, highlighting the involvement of environmental stress in the formation of spermatophyte-specific EXP genes. After comparing the GO-enriched results of coexpressed genes of group IV EXP between *A. thaliana* and *C. chingii*, the divergent functions were identified, which could be related to the phenotypic differences between ferns and seed plants (Additional file 1: Fig. S15A-B).

## Discussion

Comparing the gene expression between trophophylls and sporophylls provides insight into the developmental mechanisms underlying the diversity of fern leaves and the regulatory processes driving the formation of these phenotypically differentiated leaves [11]. In this work, we focused on *C. chingii*, a species closely related to the model fern *C. richardii* [15], which exhibits phenotypically differentiated trophophylls and sporophylls. To analyze the gene expression without a reference genome, we first generated gene models based on long-read transcriptome data of a mixed RNA pool. By combining these gene models with short-read RNA data of the separate leaf tissues (trophophylls and sporophylls sampled at two developmental stages and roots (as reference)), we were able to study the gene expression patterns. The unique data and gene models generated in this work will provide a valuable resource for future phylogenetic and molecular studies in ferns.

We identified 24,024 gene models, of which 75.25% showed a high level of similarity with the genes annotated in the *C. richardii* genome. This high similarity allowed extrapolating the intronic/exonic gene structure from *C. richardii* to *C. chingii*, which was verified for 10 randomly sampled genes by RT-PCR (Fig. 1B, Additional file 1: Fig. S2). Differential expression analysis between trophophylls and sporophylls of two developmental stages revealed a high degree of gene expression divergence between the two types of heteroblasty. Genes related to sexual reproduction were differentially expressed but only identified in the comparison between trophophyll-S1 and sporophyll-S1 (Fig. 2B). This suggests that spore development of *C. chingii* primarily occurs during the juvenile stage (i.e., S1). Notably, 20 differentially expressed genes belonged to the expansin gene family in all comparisons of heteroblastic leaves at different developmental stages. Expansin (EXP) is a plant cell wall-loosening protein. Recent studies of leaf development in seed plants revealed that overexpressing expansin activity increases leaf size and affects leaf shape by the expansion and enlargement of plant cells [48–50]. As a key regulator of cellulose/xyloglucan networks, expansin contributes to cell wall expansion [22, 51]. The dynamic expression of expansins is key to plant growth and development [22, 38], and it contributes to cell shape plasticity and organ morphology. The differential expression of EXP genes between trophophylls and sporophylls in *C. chingii* indicates that this gene family is also involved in shaping the differences in the cell wall between these two leaf types*.* RT-PCR confirmed that 12 of the EXP genes were most highly expressed in young sporophylls (Fig. 2C). Consistent with these results, we observed higher EXP content in young sporophyll compared to other tissues. This suggests that increased EXP gene expression contributes to curving sporophyll in *C. chingii.*

Having the specific gene models for ferns allowed us to reconstruct an EXP phylogeny including both ferns and seed plants. This phylogenetic tree suggests that EXP members have progressively expanded in the order of chlorophytes, bryophytes, lycophytes, ferns, and spermatophytes (Fig. 3A). Consistent with previous studies in bryophytes [52, 53], only members of the EXPA and EXPB subfamilies were identified in *C. chingii* other ferns, and lycophytes, but no members of the EXLA and EXLB subfamilies. Our phylogenetic tree also shows the presence of a vascular plant-specific group, indicating that the sequence evolution of orthologs of this gene family reflects the divergence of plant species [54, 55]. We speculate that these vascular plant-specific EXP genes may contribute to the formation of vascular systems and the divergent functions of coexpressed genes of EXP may be associated with the different phenotypes between ferns and seed plants (Additional file 1: Fig. S15). By combining the results of RNA-seq and qRT-PCR experiments, we were able to demonstrate that *EXP* genes in *C. chingii* exhibit a unique expression pattern that suggests an important role in the formation of young sporophylls and young trophophylls. To understand the regulatory network of heteromorphic leaf development in *C. chingii*, we performed gene coexpression network analysis [42, 56]. Intriguingly, shared transcription factors, lncRNA, and coexpression behavior were rarely observed between *EXP* genes of *C. chingii*, indicating that each of these *CcEXP* genes is involved in distinct regulatory networks and functions. This diversity in gene expression behavior and regulation, coupled with the expansion of a gene family, is often associated with tissue-specificity and suggests neofunctionalization [57].

Analysis of the degree to which the expression behavior of highly similar orthologs is conserved between *C. chingii* and respectively other fern species shows that, except for the high degree of expression conservation between the two related Ceratopteris species, the expression patterns of *C. chingii* genes including the EXP genes are relatively different between fern species (low EC). Given the dramatic differences in fern leaf shapes, our result suggests that expression behavior has largely diverged, particularly within fern species. This low degree of coexpression conservation between EXP genes of different species was further confirmed by analyzing the degree to which interactions in the coexpression networks of the EXP genes were conserved across species. Coexpression interactions were mainly conserved between *CcEXP* and *CrEXP* genes, as well as between *CcEXP* and *AtEXP* genes. Conclusively, the significant degree of sub and neofunctionalization observed suggests that EXP genes are associated with the formation of heteroblasty, which exhibits phenotypic differences between species.

## Methods

### Plant sample collection and RNA isolation

The wild *Ceratopteris chingii* plants were collected from the Zhangdu Lake at Wuhan (114° 31′ E, 30° 52′ N) and cultivated in the greenhouse in the Wuhan botanical garden. A single root sample was collected. We harvested whole leaves of both trophophylls and sporophylls at two developmental stages. Stage 1 (S1, young) had a blade length of 2~3 cm, while stage 2 (S2, old) had a blade length of 5~6 cm. The samples were washed with distilled water and then frozen in liquid nitrogen. Total RNA was extracted from each sample using the RNAprep Plant Kit (TIANGEN). RNA concentration and integrity were examined using the Qubit RNA Assay Kit in a Qubit 2.0 Fluorometer (Life Technologies) and the Agilent 2100 Bioanalyzer (Agilent Technologies).

### PacBio Iso-Seq of a mixed RNA sample

Equal quantities of RNA were extracted from five tissue samples, including root, trophophyll-S1, sporophyll-S1, trophophyll-S2, and sporophyll-S2, for the construction of a PacBio cDNA library. Briefly, 1 μg of RNA from the pooled RNA sample was reverse transcribed using the Clontech SMARTer polymerase chain reaction (PCR) cDNA Synthesis Kit and oligo (dT) to generate the first-strand cDNA. The second strand was then synthesized and amplified with the 5′ PCR primer to obtain full-length cDNA with barcodes. Size selection was carried out using the BluePippin Size Selection System protocol, with two size bins for the mixed sample: 1–4 kb and > 4 kb. Finally, the cDNA library was sequenced on the PacBio Sequal platform using a SMRT cell.

### Iso-Seq data analysis

The standard Iso-Seq protocol (SMRTlink 4.0) was used to process raw sequencing data (81.81Gb) with min-length > 200 and min-read-score > 0.75. A total of 669,146 circular consensus sequencing (CCS) reads were generated from subread sequences with accuracy > 0.8 and further classified into full-length non-chimeric reads or non-full-length reads depending on whether the 5′ primer, 3′ primer, and a poly-A tail signal preceding the 3′ prime were present. Full-length reads were clustered and assembled into consensus sequences with iterative clustering for error correction (ICE). Subsequently, polished consensus reads were obtained by removing sequence errors using the non-full-length reads and Illumina RNA-seq data with Arrow and LoRDEC. To obtain the final non-redundant high-quality full-length transcripts, the error-corrected full-length polished consensus transcripts were merged and redundancy removed using CD-HIT with the following parameters: -c 0.99 -aS 0.99 -AS 30.

### Gene structural annotation

Due to the lack of a reference genome for *C. chingii*, we processed non-redundant high-quality full-length transcripts were processed using the Coding GENome Reconstruction Tool (Cogent, https://github.com/Magdoll/Cogent). In summary, Cogent clusters transcripts into families based on their *k*-mer similarity and subsequently converts each transcript family into one or several unique transcript models (UniTransModels, further regarded as gene models) using a De Bruijn graph-based method. To improve the accuracy of the UniTransModels, the non-redundant high-quality full-length transcripts were mapped to the chromosome-level genome assembly of *C. richardii* using GMAP with -min-trimmed-coverage=0.85 --min-identity=0.9. If the transcripts from a *C. chingii* gene model were mapped to a unique gene locus on the *C. richardii* genome, the *C. chingii* gene model was considered a high-confident gene. A *C. chingii* gene model was considered a low-confident gene if a gene model’s specific transcript (1) maps ambiguously to multiple gene loci in *C. richardii*, (2) partially maps to a unique gene locus in *C. richardii*, or (3) partially maps to multiple gene loci in *C. richardii*. Ten randomly selected splice events derived from 10 high-confident gene models were examined by RT-PCR, using the primers listed in Additional file 2: Table S6. RNA of each sample (1 μg) was reverse transcribed into cDNA and used for PCR amplification. PCR fragments were tested in 1% agarose gel.

### Gene functional annotation and long non-coding RNA identification

For the functional annotation of the gene models, we used the following databases: Gene Ontology (GO), Kyoto Encyclopedia of Genes and Genomes (KEGG), Swiss-Port, Pfam, and NCBI Non-redundant Protein (Nr). To analyze the enrichment of gene functions, we employed TBtools. To identify which of the gene models corresponded to transcription factors (TF), we used PlantTFDB/Itak. The coding potential of the gene models was evaluated by the following four tools: Coding Potential Calculator (CPC2), Coding-Non-Coding Index (CNCI), Pfam-scan, and Coding Potential Assessment Tool (CPAT). For reliable identification of non-coding RNAs, we considered those with sequence lengths longer than 200 nt and an average FPKM over 0.1. The genes targeted by these lncRNAs were predicted using the LncTar (http://www.cuilab.cn/lnctar) and RPISeq (http://pridb.gdcb.iastate.edu/RPISeq/).

### RNA-seq and transcriptome analysis

For each sample, the cDNA sequencing library was constructed and sequenced on the Illumina platform following the standard protocol. The reads were then trimmed and filtered by removing adapters and low-quality reads. Afterward, the trimmed and filtered reads were error-corrected to eventually obtain the polished consensus full-length sequences mentioned earlier. To estimate the gene expression, the trimmed and filtered reads were mapped to the obtained gene models using Hisat2. The expression level of each gene model (measured in fragments per kilobase of exon model per million mapped fragments (FPKM)) in each of the different samples was estimated using StringTie. Differentially expressed genes (DEGs) were identified using DEseq2, with a fold change (FC) greater than 2 and a false discovery rate (FDR) less than 0.05 as the criteria. Furthermore, all 12 *CcEXP* genes showing tissue-specific expression in sporophyll-S1 and trophophyll-S1 were selected for qRT-PCR experiments. However, due to the high sequence similarity of two pairs of genes (i.e., respectively *CcFL01488*-*CcFL01489* and *CcFL08912*-*CcFL08913*), we selected one representative of each pair only. As a result, qRT-PCR experiments were conducted on ten uniquely differentially expressed *CcEXP* genes. The experiments for the five RNA-seq samples were performed under the following conditions: 95 °C for 30 s; 40 cycles of 95 °C for 5 s, 60 °C for 30 s, and 72 °C for 15s; and 95 °C for 10 s. Relative expression levels were calculated using the 2^−ΔΔCt^ method, with the *GAPDH* gene used as the internal standard. The primers for these 10 DEGs can be found in Additional file 2: Table S7.

### Expansin gene identification and phylogenetic analysis

Homologous genes were identified with Orthofinder [58] with genomic data from a range of taxa sampled across the plant tree of life, including (a) four green algae: *Chlamydomonas reinhardtii* [59], *Klebsormidium flaccidum* [60], *Mesotaenium endlicherianum* [61], and *Spirogloea muscicola* [61]; (b) three bryophytes: *Marchantia polymorpha* [62], *Physcomitrium patens* [63], and *Sphagnum fallax* [64]; (c) two lycophytes: *Isoetes taiwanensis* [65] and *Selaginella moellendorffii* [66]; (d) four ferns: *Azolla filiculoides* [29], *C. chingii* (this study), *C. richardii* [15], and *Salvinia cucullate* [29]; and (e) six seed plants: *Amborella trichopoda* [67], *Arabidopsis thaliana* (http://www.arabidopsis.org), *Brachypodium distachyon* [68], *Ceratophyllum demersum* [69], *Cycas panzhihuaensis* [70], and *Pica abies* [71]. All reported expansin protein sequences of *A. thaliana* were downloaded from TAIR11 (http://www.arabidopsis.org). The *A. thaliana* expansin (*AtEXPs*) protein sequences were mapped to each of the genomic datasets listed above using BLAST with an *e*-value < 1e−5. The mapped genes, along with the genes in each of the investigated genomes that belong to the same orthologous group as the *AtEXPs*, were identified as candidate orthologs. Candidate genes were further filtered by only retaining those that had both conserved domains: DPBB-1 and Expansin_C, characteristic for expansin proteins. This resulted in 538 expansin genes extracted from all previously mentioned genomes, except from *Chlamydomonas reinhardtii*. As for the latter species, the candidates did not contain an Expansin_C domain, and they were not retained. Filtered candidate proteins were aligned using MAFFT v7.453, and positions with more than 95% gaps were removed using Phyutility v.2.7.1 (-clean 0.05). A maximum likelihood (ML) tree of selected genes was constructed using IQTree with 1000 bootstrap replicates and an MFP model. Gene subfamilies were delineated, based on the position of outgroups and previously identified expansins of *A. thaliana*.

### Coexpression network analysis of EXP genes in C. chingii, C. richardii, and A. thaliana

The expression level of each gene model was estimated for each of the different tissues (see gene expression matrix). The gene coexpression network was built as follows: (1) non-expressed genes with an average FPKM < 0.1 and constitutively expressed genes with a coefficient of variation (CV) < 0.5 were removed, while the expression level of the remaining genes was log-transformed with log_2_; (2) the correlation between an expansin gene and any other gene was evaluated using Spearman correlation; and (3) only pairwise correlations > |0.95| and with a *p*-value < 1e−5 were retained to construct a co-expression network.

To generate a list of the most reliable coexpression interactions, we calculated the mutual rank (MR) of pairwise correlations:

$$\mathrm{MR}\left(ab\right)=\sqrt{(\mathrm{Rank}(a\to b)\times \mathrm{Rank}(b\to a)}$$

Only coexpression interactions identified by the Spearman correlation that were also in the top 1% of the interactions identified by the MR were considered reliable.

To perform a comparative analysis of gene coexpression networks across species, we used *C. richardii* as a species closely related to *C. chingii* and *A. thaliana* as a representative of seed plants (outgroup). A total of 12 RNA-seq *C. richardii* samples (4 trophophylls, 4 sporophylls, and 4 roots) and 21 RNA-seq *A. thaliana* samples (8 mature leaves, 8 young leaves, and 5 roots) were downloaded from previous studies (Additional file 2: Table S5). For each sample, the filtered and trimmed reads were mapped to their respective reference genomes using Hisat2. StringTie was used to estimate the expression levels in the different tissues. The EXP gene coexpression networks in *C. richardii* and *A. thaliana* were constructed in the same way as the one of *C. chingii* (see above).

### Comparative analysis of gene expression behavior between orthologs

To compare the expression divergence of orthologous genes between *C. chingii* and related fern and lycophyte species, we downloaded, in addition to the RNA of *C. richardii* (see above), also the RNA-seq samples of leaf and root in *S. cucullata*, *I. taiwanensis*, and *S. moellendorffii* from the NCBI SRA database (Additional file 2: Table S5). For each species, the clean reads of the different tissue samples were mapped to their respective reference genome using Hisat2 [72], and gene expression matrices were constructed. For each *C. chingii* gene, we identified its closest relative in other species, i.e., the ortholog that is most similar to the *C. chingii* gene using the following criteria: (1) orthologous genes should belong to the same orthologous group as the considered *C. chingii* genes (identified by the Orthofinder) and (2) should be the best hit of BLASTP with e-value < 1e−5 in bidirectional comparisons of two species. We used these pairs of “highly similar orthologs” to perform the comparative expression analysis.

To cope with the difference in the number and type of tissue samples that were available for each of the compared species, we used the iterative algorithm from Tirosh et al. [47] to assess the expression conservation of the identified orthologous gene pairs. For each species, a gene expression matrix was built that lists, in the rows, the genes in the n orthologous gene pairs ranked in the same order and, in the columns, the profiled conditions in each of the species (${E}_{g,x}^{\mathrm{SpeciesA}}$, ${E}_{g,y}^{\mathrm{SpeciesB}}$, where *g* = 1..*n*). Each of these expression matrices is converted to a corresponding correlation matrix (${R}_{g,g}^{\mathrm{SpeciesA}}$, ${R}_{g,g}^{\mathrm{SpeciesB}}$, where *g* = 1..*n*) by calculating the Pearson correlation coefficient (PCC) between the gene expression profiles (*n* × *n* matrix). The initial estimation of expression conservation (EC_0_) is obtained by comparing the equivalent rows of the two correlation matrices (i.e., comparing the degree to which a pair of orthologs shares similar correlations with other orthologs pairs, i.e., a correlation of the vector in each matrix that contains the correlation coefficient of the gene *i* with any other gene *g* in the matrix).

$${\mathrm{EC}}_{0}(i)=\mathrm{PPC}({R}_{i,g}^{\mathrm{SpeciesA}}, {R}_{i,g}^{\mathrm{SpeciesB}})$$

However, when comparing a pair of orthologs, one would like to focus on their correlations with other orthologous pairs of which the expression has been conserved as this allows compensating for the differences in the profiled conditions. The iterative algorithm of expression conservation (EC) therefore calculates a weighted correlation, where the weight assigned for correlation with each gene is provided by the EC of that gene from the previous iteration. Genes with negative weights are excluded from the calculation

$${\mathrm{EC}}_{k}(i)=\mathrm{PPCw}({R}_{i,{g}{\prime}}^{\mathrm{SpeciesA}}, {R}_{i,{g}{\prime}}^{\mathrm{SpeciesB}})$$

where PPCw(*x*,*y*) =$$\frac{\sum {w}_{i}\left({x}_{i}-\overline{x }\right)\left({y}_{i}-\overline{y }\right)}{\sqrt{\sum {w}_{i}({x}_{i}-\overline{x }{)}^{2}}\sum {w}_{i}({y}_{i}-\overline{y }{)}^{2}} , {w}_{i}={\mathrm{EC}}_{k-1}\left(i\right) , {g}{\prime}=\left\{l \in g \right|{\mathrm{EC}}_{k-1}>0\}$$

The criteria for the end of the iteration are as follows:

$$\sum_{i\in g}[{\mathrm{EC}}_{k}\left(i\right)-{\mathrm{EC}}_{k-1}\left(i\right){]}^{2}<0.1$$

### Expansin content measurements

The amount of expansin was measured in trophophyll-S1, sporophyll-S1, trophophyll-S2, sporophyll-S2, and roots using five repeats for each tissue type. Approximately 0.5 g fresh weight tissues were cleaned, blotted dry, and snapfrozen in liquid nitrogen. Tissue samples were ground with 2-mL ice-cold extraction buffer with 50 mM Tris, 0.1 mM EDTA, and 15 mM MgCl_2_ at PH 8 plus 10% glycerol. The homogenate of tissue samples was centrifuged at 4 °C for 15 min, and the supernatant was obtained and stored at − 80 °C. The expansin content in the supernatant was measured using the Plant EXPANSIN Elisa Kit (Zhen Ke Biological Technology, Shanghai) following the operating instructions. .

### Yeast two-hybrid (Y2H)

Y2H assays were performed using the Matchmaker Gold Two-Hybrid system (Clontech). The full-length CDS fragments of *CcFL01489*, *CcFL03547*, *CcFL06843*, *CcFL09745*, and *CcFL13362* were amplified from *C. chingii* cDNA. These fragments were then fused as BamHI/EcoRI fragments into pGKBT7 (BD) and pGADT7 (AD) using the Basic Seamless Cloning and Assembly Kit (TransGen, Beijing, China) to generate *CcFL01489*-BD (bait vector), *CcFL03547*-AD (prey vector), *CcFL06843*-AD (prey vector), *CcFL09745*-AD (prey vector), and *CcFL13362*-AD (prey vector), respectively. The bait vector and prey vector were co-transformed into the Y2H Gold strain performed as previously described [73], with the empty vector as a negative control. Positive transformants were selected on SD/-Trp/-Leu dropout medium and further screened on SD/-Leu/-Trp/-His/-Ade + X-α-gal (40 μg mL^−1^) dropout medium. The cultures were then incubated at 30 °C for 3 days. The primers used in the Y2H assay are listed in Additional file 2: Table S8.

### Supplementary Information

**Additional file 1.** **Fig. S1.** Density distribution of raw reads and full-length non-chimeric reads obtained by PacBio Iso-seq. **Fig. S2.** RT-PCR validation of nine high-confident gene models. **Fig. S3.** The UpSet plot summarizes the presence of genes in five databases. **Fig. S4.** The pie diagrams showing the number of transcription factors in different families. **Fig. S5.** GO enrichment analysis of differentially expressed genes in the comparisons of sporophyll-S1 vs trophophyll-S2, sporophyll-S2 vs trophophyll-S1, and sporophyll-S2 vs trophophyll-S2. **Fig. S6.** Heatmap showing the differential and non-differential expression of the expansin genes in different pairwise comparisons as obtained from transcriptome analysis. **Fig. S7.** Phylogenetic tree of expansins in *C. chingii* and *A. thaliana*, built with Maximum likelihood. **Fig. S8.** Analysis of conserved domains of the *CcEXP* proteins. **Fig. S9.** Phylogenetic profiling of four groups of expansin genes in 19 species. **Fig. S10.** Bar plots showing the differentially expressed expansin genes in pteridophyte-specific groups. **Fig. S11.** GO enrichment analysis of genes that were identified to be significantly coexpressed with *CcEXP* genes. **Fig. S12.** Heatmap showing the member number of transcription factors in different families that are coexpressed with at least one *CcEXP* gene in the four groups. **Fig. S13.** Yeast two-hybrid assay showing how *CcFL01489* interacts with three transcription factor (*CcFL09745*, *CcFL03547*, and *CcFL06843*). **Fig. S14.** Gene coexpression networks of *CrEXP* (A) and *AtEXP* (B) genes of the different groups. **Fig. S15.** GO enrichment analysis of genes that were identified to be significantly coexpressed with *AtEXP* (A) and *CcEXP* (B) genes in groupIV.

**Additional file 2.** **Table S1.** EXP genes in *C. chingii*. **Table S2.** Topology of EXP gene tree of 19 species. **Table S3.** Correlation coefficient of FPKM and relative expression in *CcEXP* genes. **Table S4.** Summer of lncRNA targeted genes. **Table S5.** Summary of the RNA-Seq samples in this study. Table S6. Summary of the RT-PCR primer for validating the splicing events. **Table S7.** Summary of the qRT-PCR primer for expansin genes. **Table S8.** Summer of Yeast two-hybrid primer for *CcFL01489* genes and its coexpressed transcription factors.

## Data Availability

The PacBio full-length sequencing dataset generated for this work is accessible through NCBI Sequence Read Archive (SRA) under accession number PRJNA924672 [74]. The Illumina RNA-seq dataset is accessible through NCBI SRA accession number PRJNA924581 [75].

## Abbreviations

BUSCO: Benchmarking universal single-copy orthologs
DEG: Differentially expressed gene
EC: Expression conservation
EXP: Expansin
FLNCs: Full-length non-chimeric reads
FPKM: Fragments per kilobase per million mapped reads
GO: Gene Ontology
ICE: Iterative clustering for error correction
KEGG: Kyoto Encyclopedia of Genes and Genomes
KOG: Eukaryotic Orthologous Groups
MEME: Multiple expectation maximization for motif elicitation
ML: Maximum likelihood
RT-PCR: Reverse transcription-polymerase chain reaction
qRT-PCR: Real-time quantitative reverse transcription-polymerase chain reaction
