# Supplementary material for: Expression divergence of expansin genes drive the heteroblasty in Ceratopteris chingii
Source: BMC Biol. 2023 Nov 6;21:244. doi: 10.1186/s12915-023-01743-7 (PMC10626718; doi:10.1186/s12915-023-01743-7)

Supplementary Figures:

Figure S1. Density distribution of raw reads and full-length non-chimeric reads (FLNCs) obtained by PacBio Iso-seq.

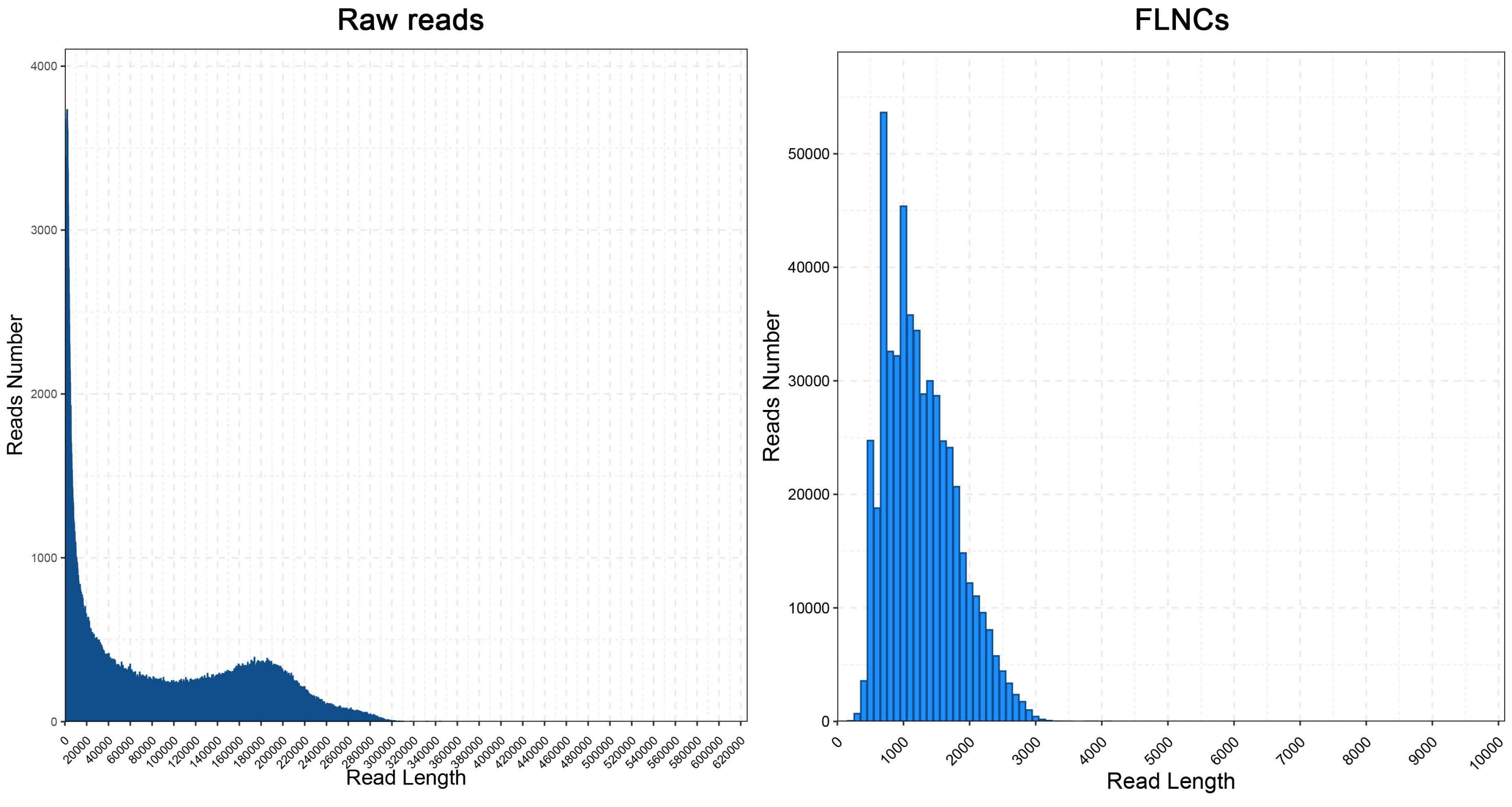

Figure S2. RT-PCR validation of nine high-confident gene models (A-I). The whole isoforms of nine *C. chingii* gene models were respectively mapped to *C. richardii* using BLAT and further clustered in nine gene regions. The arrows show the loci of the PCR primers (F, forward, and R, reverse) on the last isoform. The RT-PCR amplifications in each species among tissues are shown in the right panel.

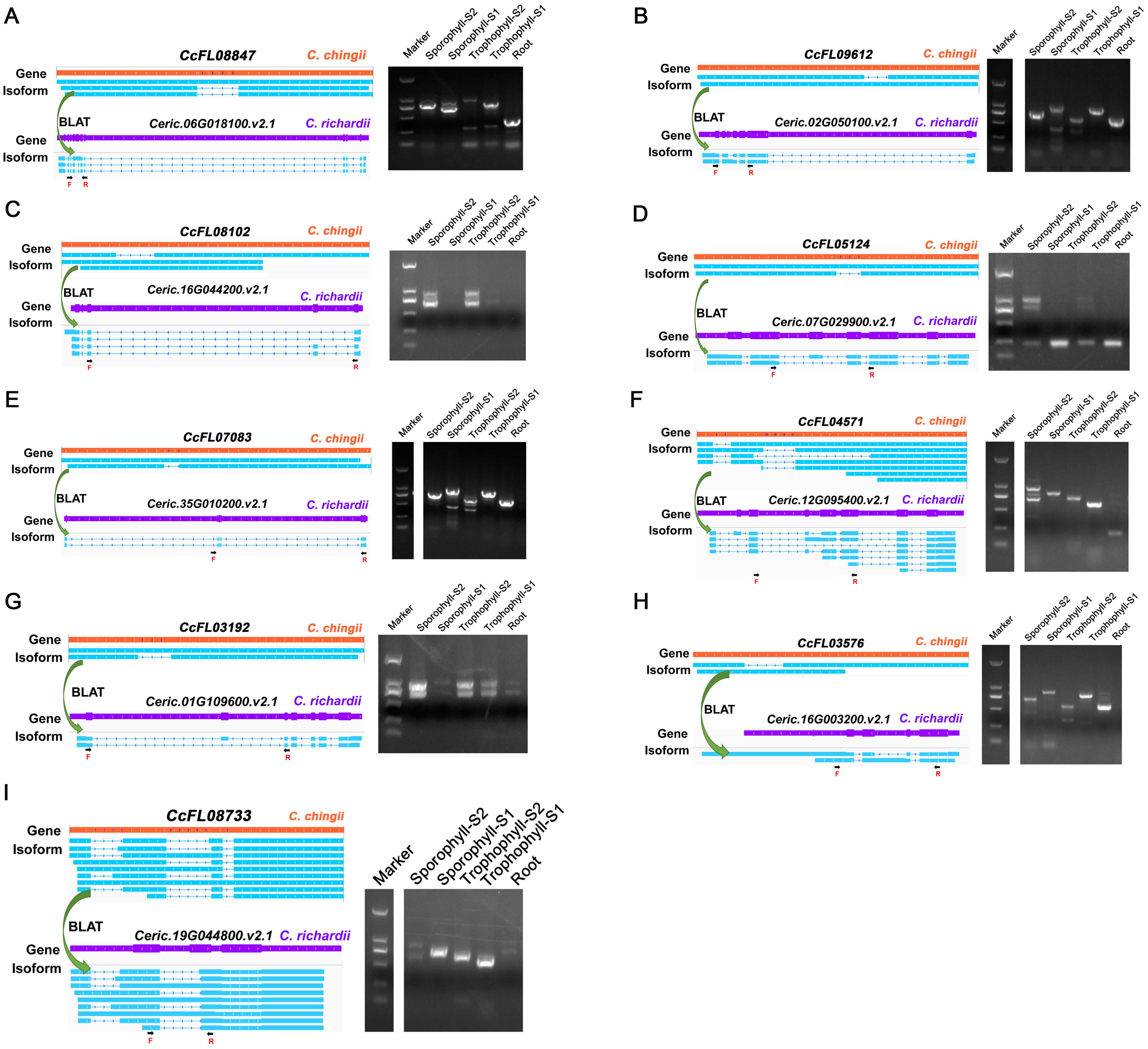

Figure S3. The UpSet plot summarizes the presence of genes in five databases. The bottom left horizontal bar graph shows the total number of genes in per database. The circles in each panel's matrix represent the unique and common parts in Venn diagram sections (unique and overlapping genes). Connected circles indicate a certain intersection of genes between databases. The top bar graph in each panel summarizes the number of gene for each unique or overlapping combination.

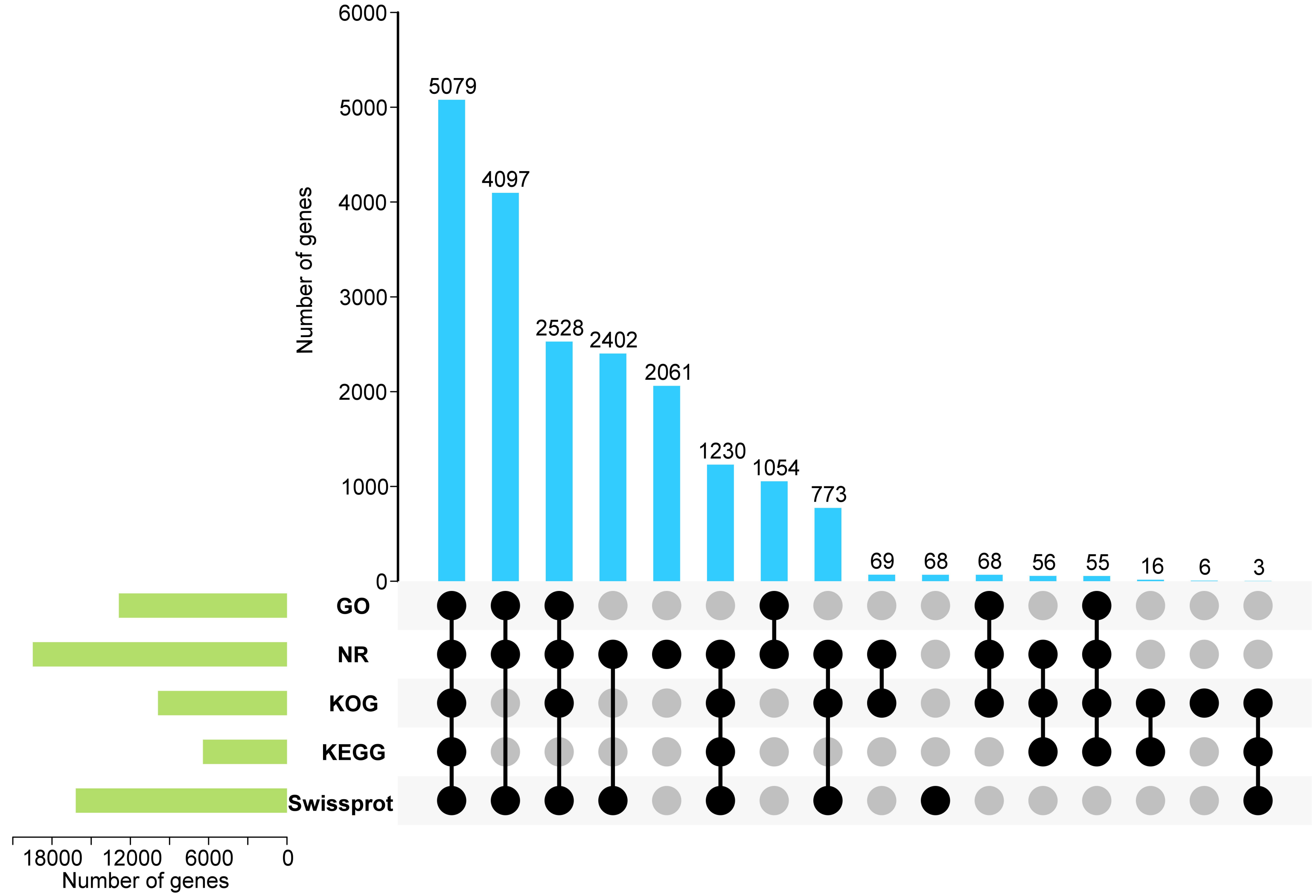

Figure S4. The pie diagrams showing the number of transcription factors in different families.

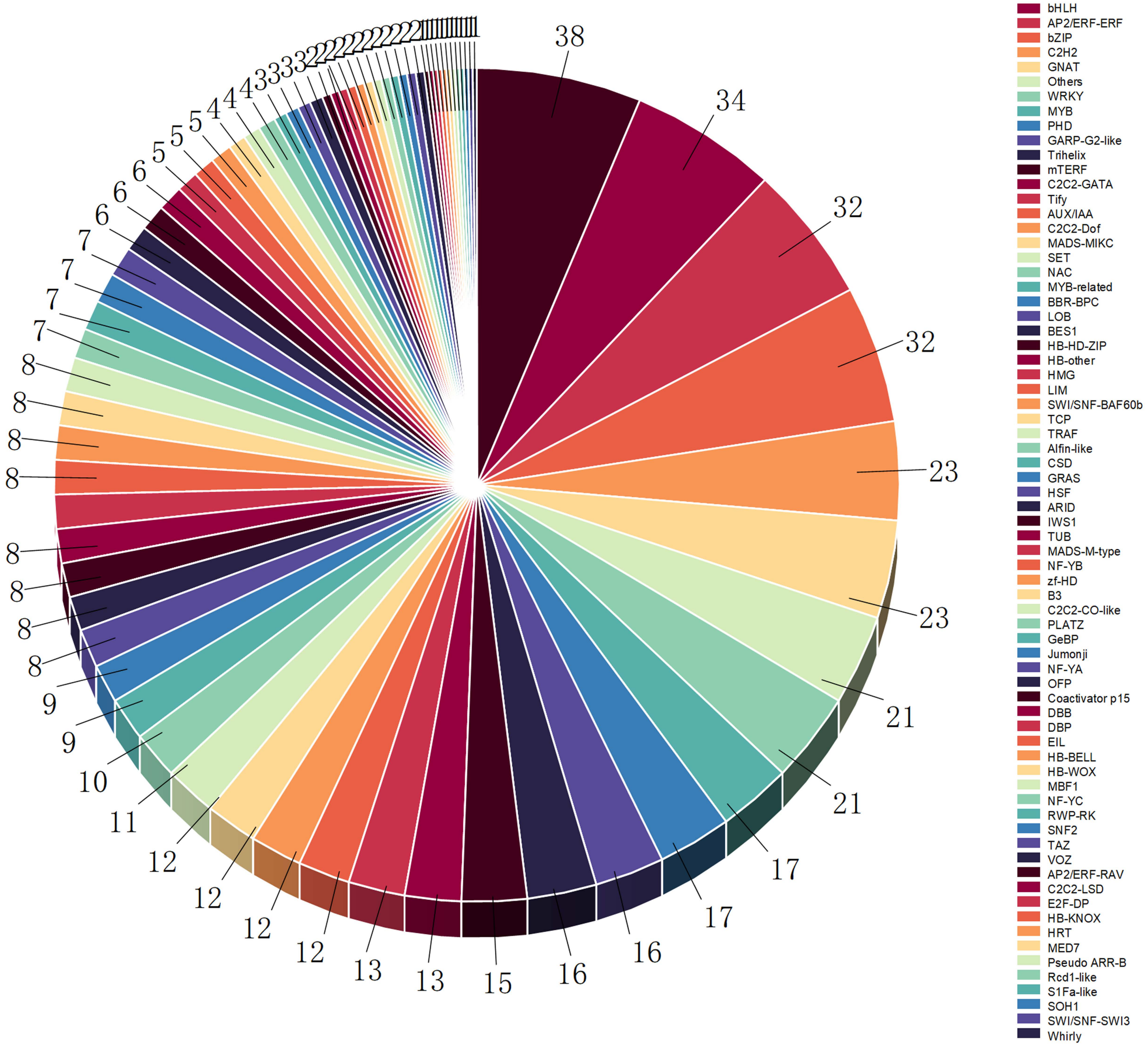

Figure S5. GO enrichment analysis of differentially expressed genes in the comparisons of sporophyll-S1 vs trophophyll-S2, sporophyll-S2 vs trophophyll-S1, and sporophyll-S2 vs trophophyll-S2.

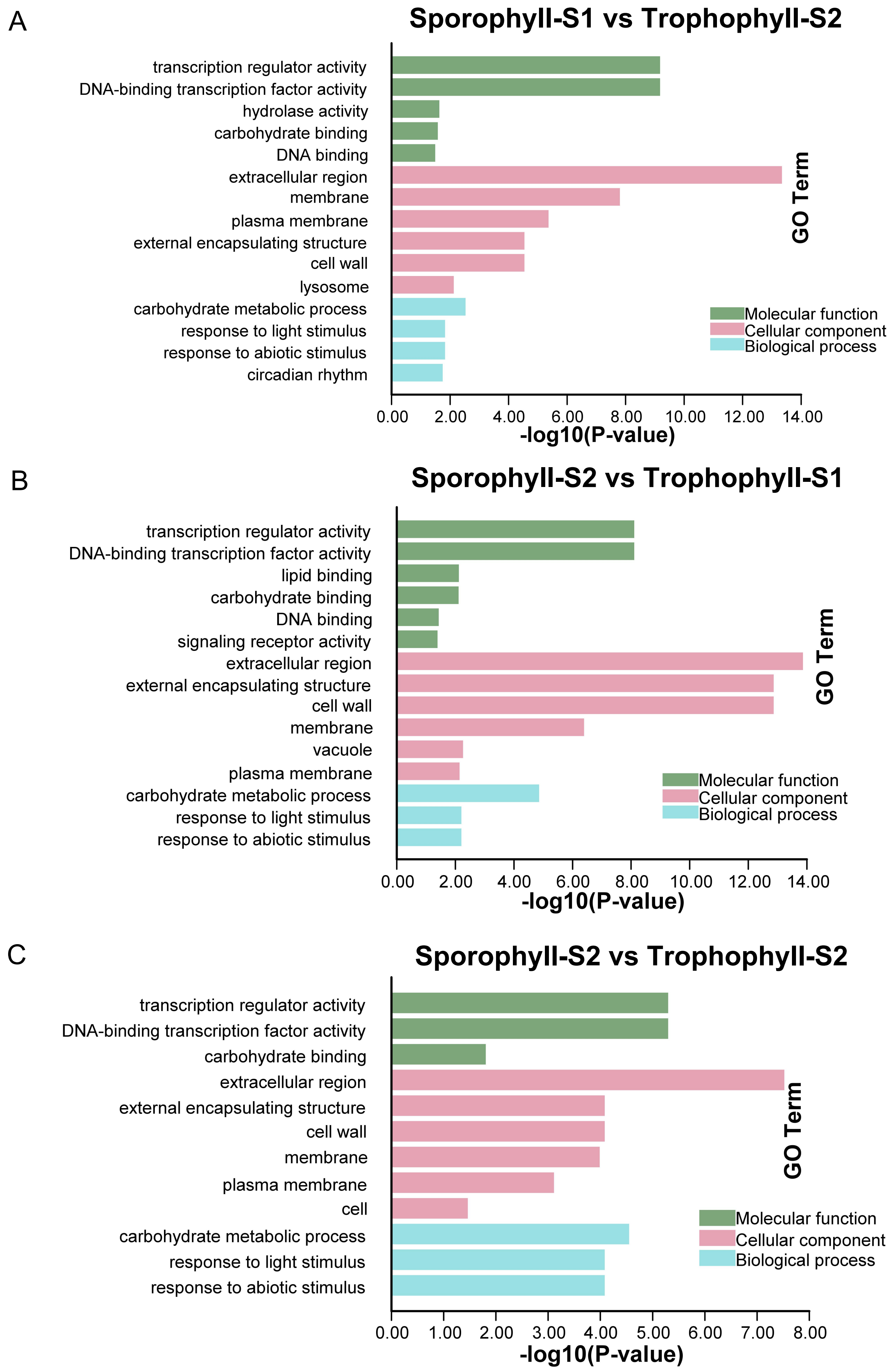

Figure S6. Heatmap showing the differential and non-differential expression of the expansin genes in different pairwise comparisons as obtained from transcriptome analysis.

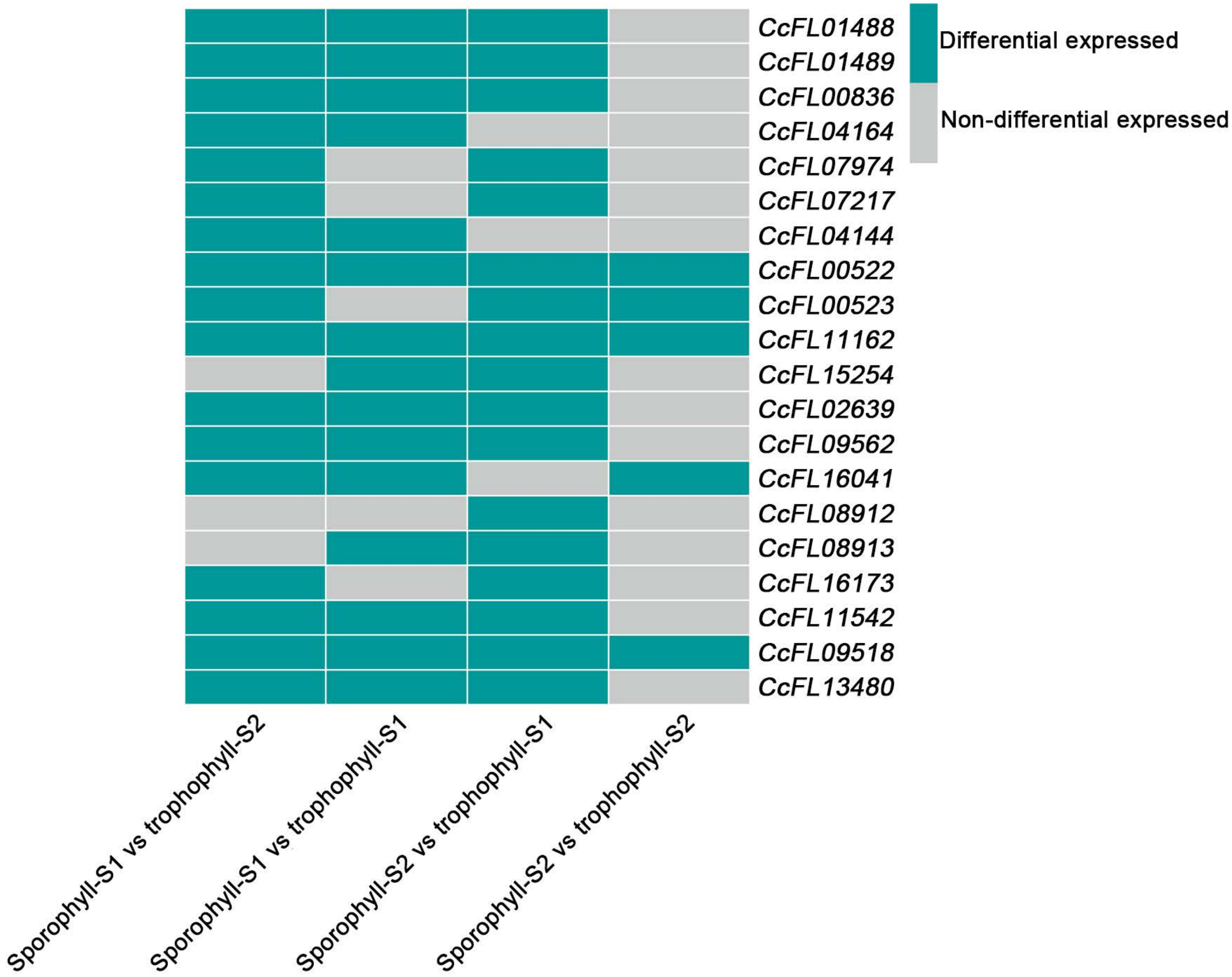

Figure S7. Phylogenetic tree of expansins in *C. chingii* and *A. thaliana*, built with Maximum likelihood.

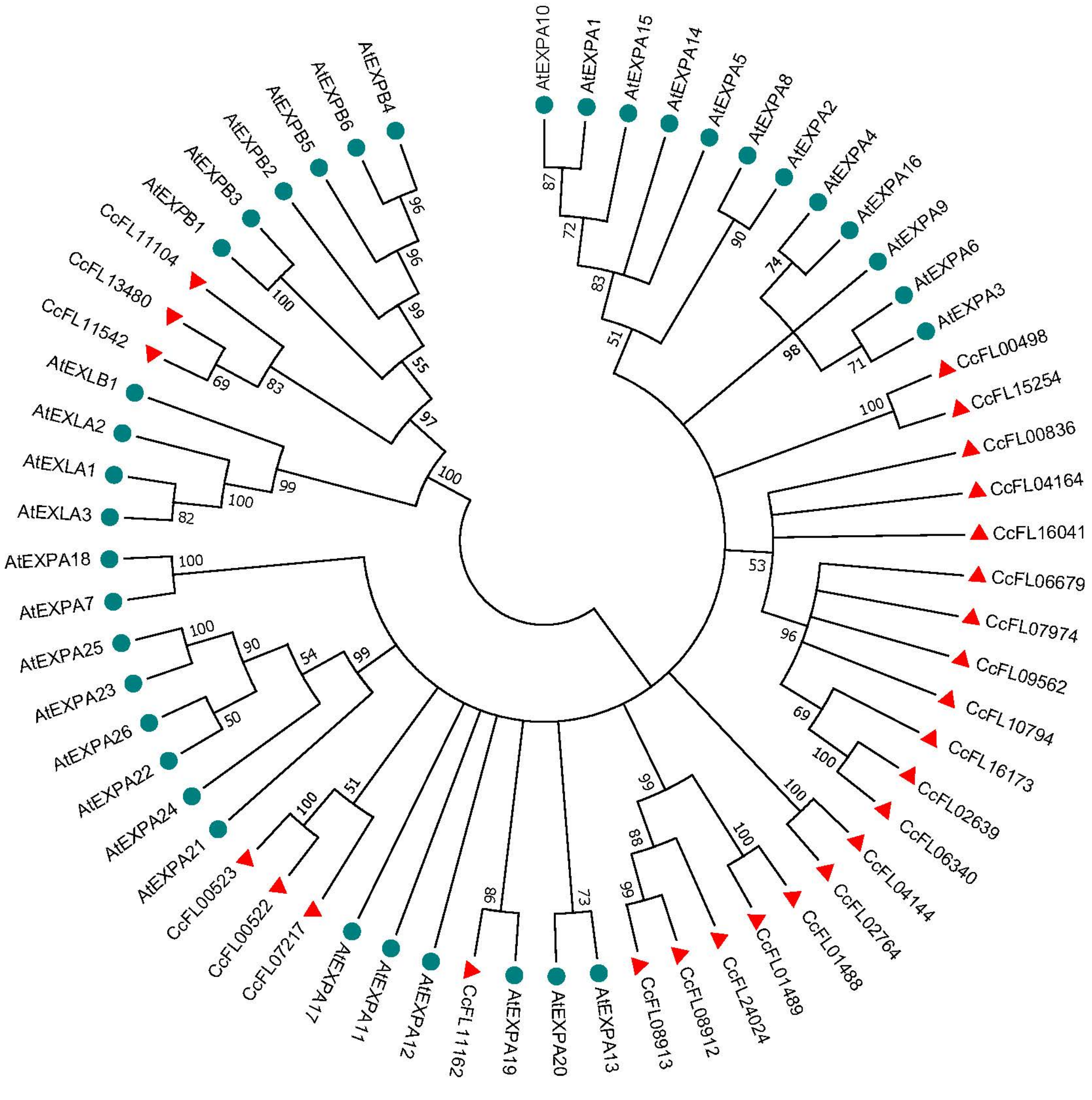

Figure S8. Analysis of conserved domains of the *CcEXP* proteins. Boxes with different colours represent different conserved motifs identified by the MEME.

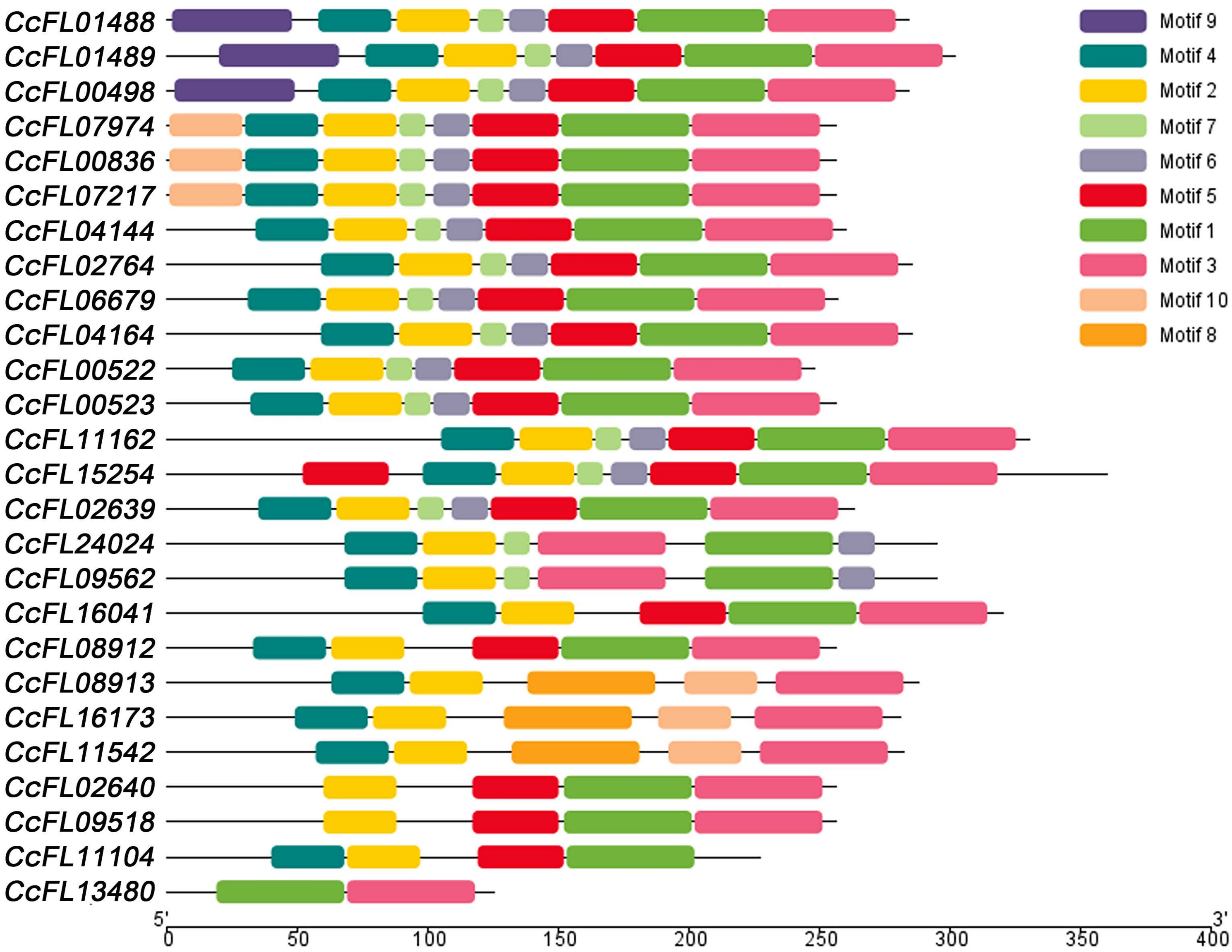

Figure S9. Phylogenetic profiling of four groups of expansin genes in 19 species.

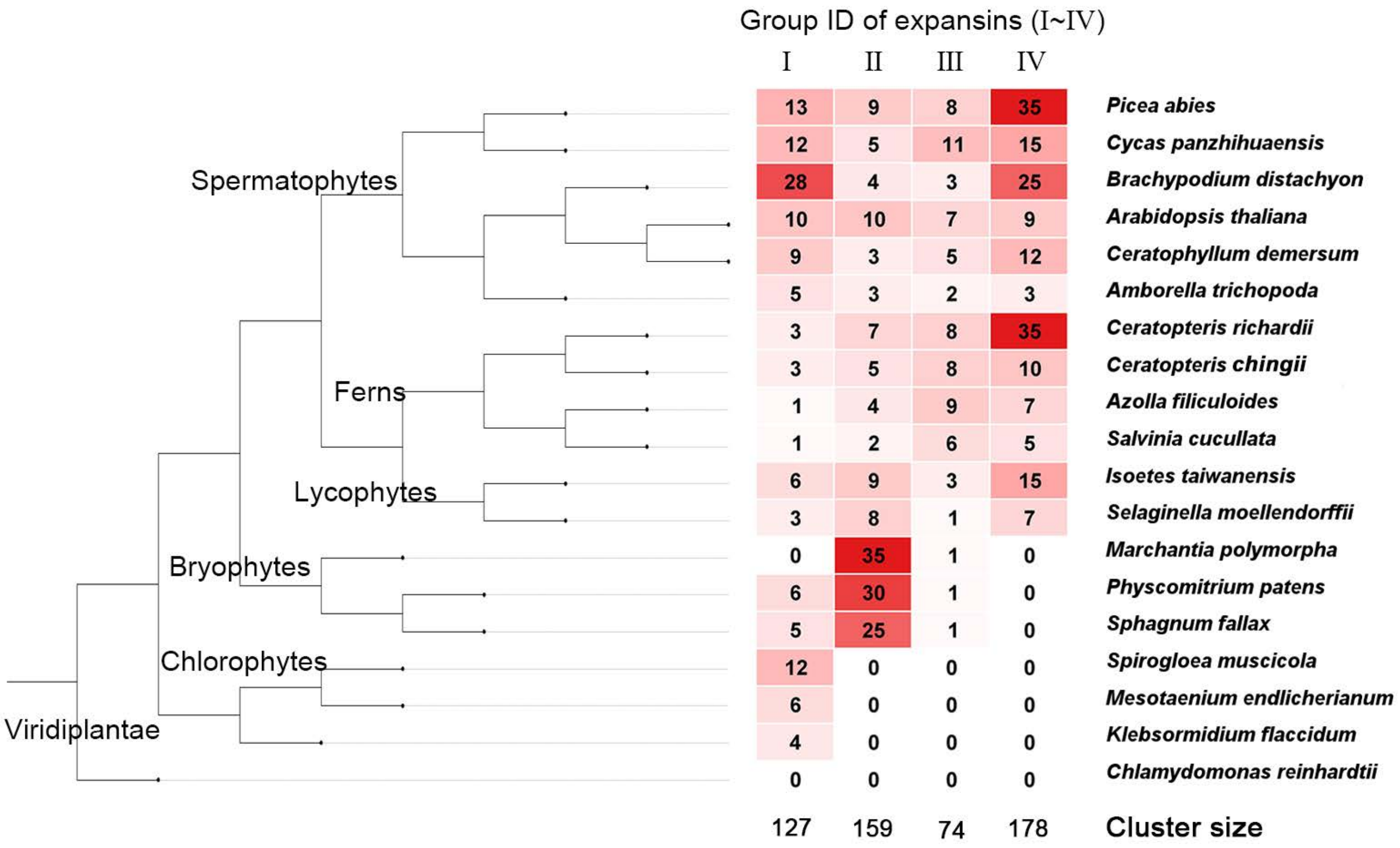

Figure S10. Bar plots showing the differentially expressed expansin genes in pteridophyte-specific groups. Red: qRT-PCR results; green: RNA-seq results.

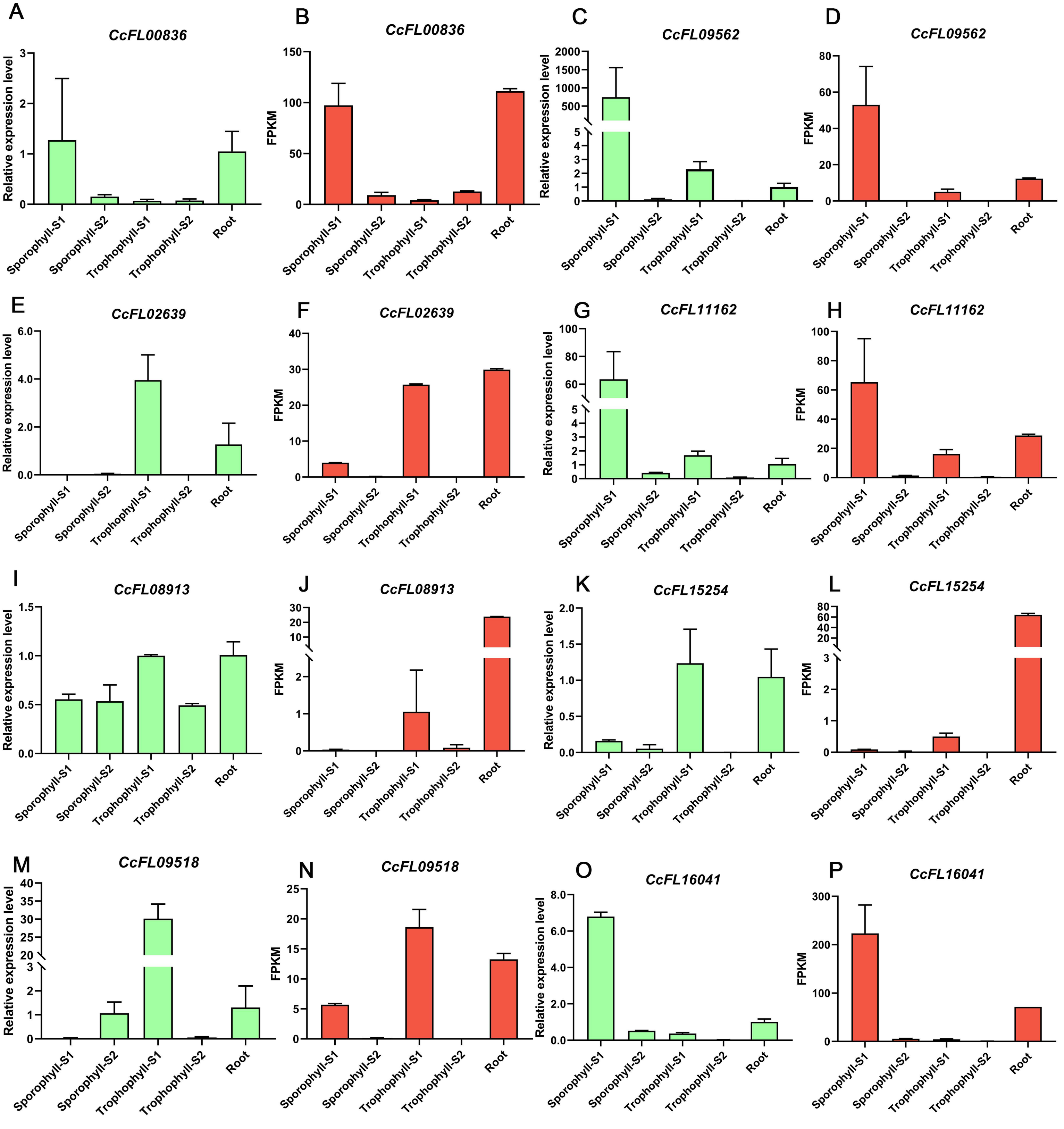

Figure S11. GO enrichment analysis of genes that were identified to be significantly coexpressed with *CcEXP* genes.

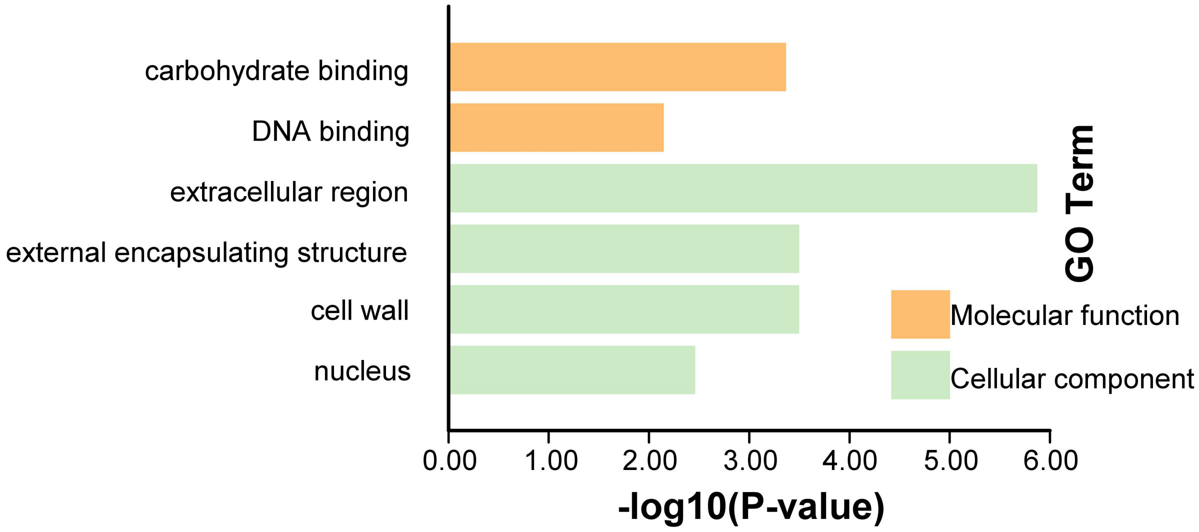

Figure S12. Heatmap showing the member number of transcription factors in different families that are coexpressed with at least one *CcEXP* gene in the four groups.

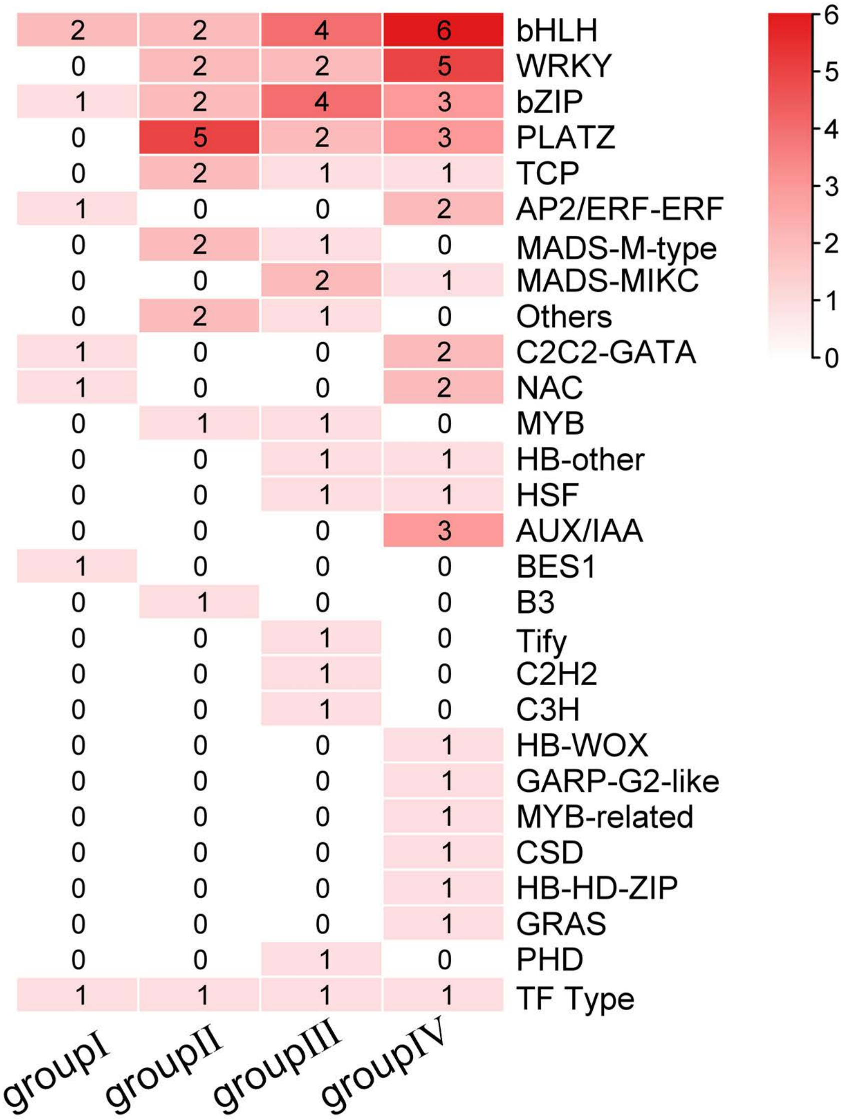

Figure S13. Yeast two-hybrid assay showing how CcFL01489 interacts with three transcription factor (CcFL09745, CcFL03547, and CcFL06843). Empty vector pGKBT7 (BD) and pGADT7 (AD) as the negative control. Recombinant plasmids were transformed into Y2H gold yeast strain and grown on selection medium at 30 ℃ for 3 days

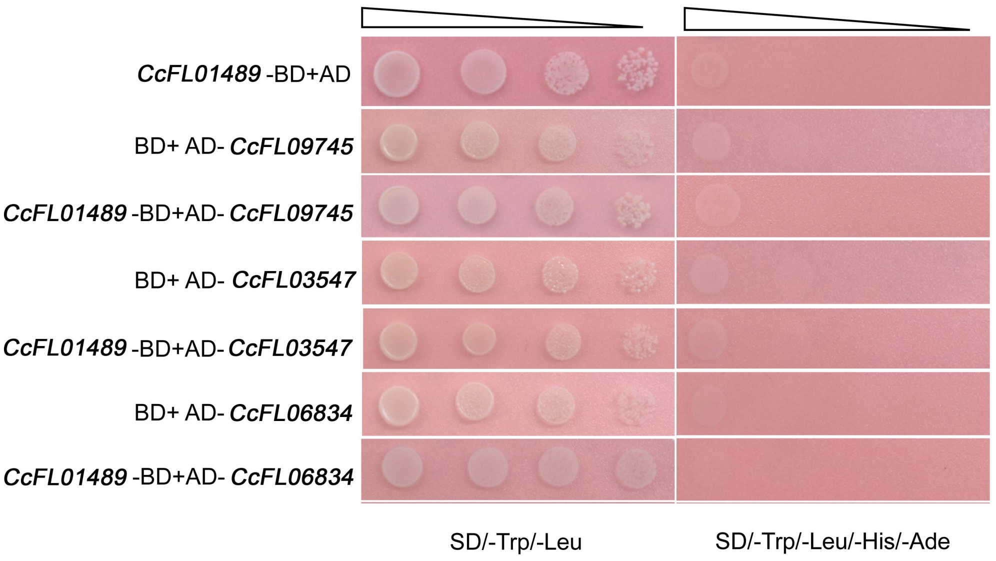

Figure S14. Gene coexpression networks of *CrEXP* (A) and AtEXP (B) genes of the different groups. The hexagons represent the EXP genes, the rhombus represent the transcription factors and the circles represent the coexpressed genes. The red lines link the significantly (r > 0.95) positive coexpression interactions, and the blue lines link the significantly (r < -0.95) negative coexpression interactions.

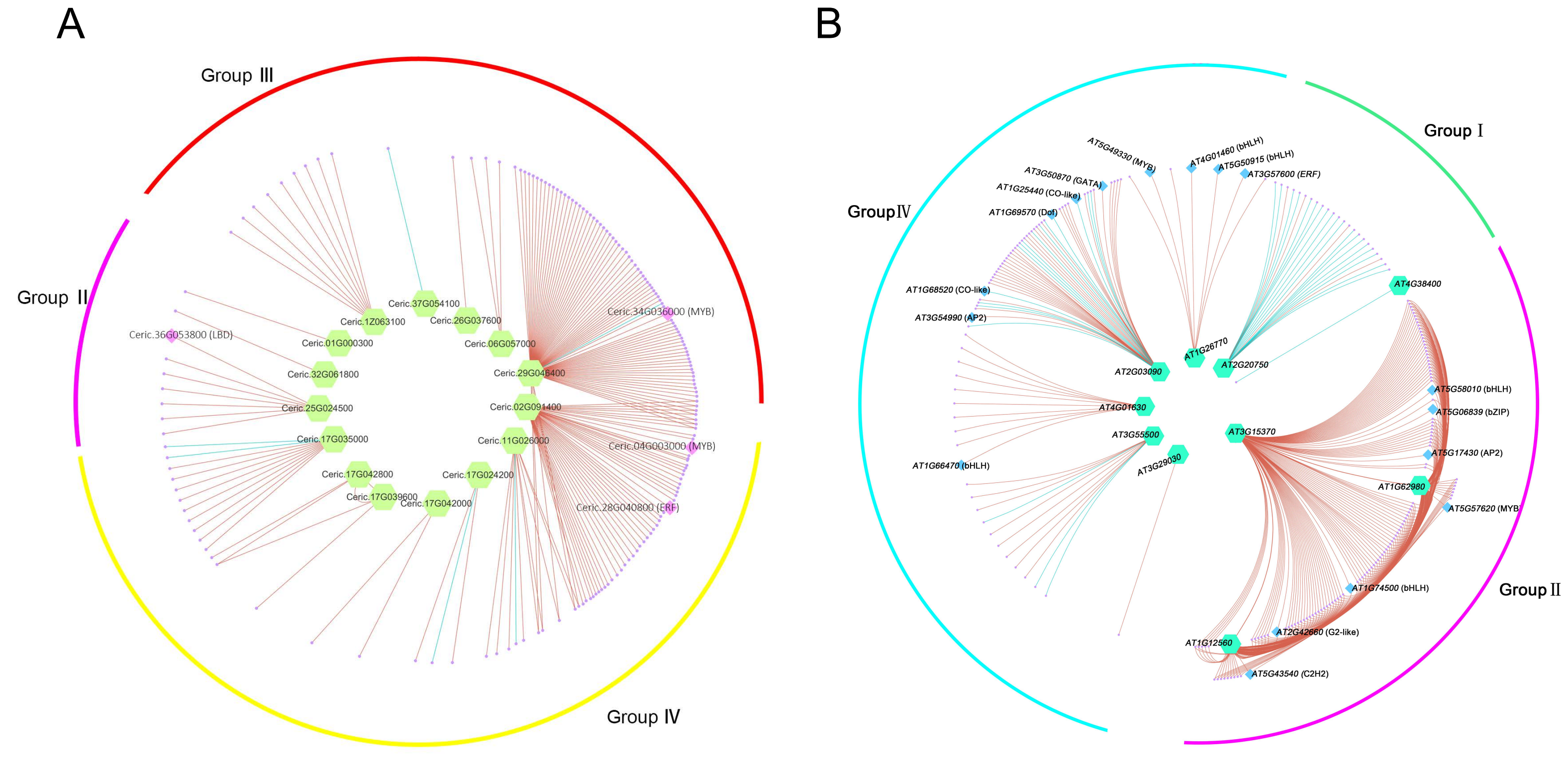

Figure S15. GO enrichment analysis of genes that were identified to be significantly coexpressed with *AtEXP* (A) and *CcEXP* (B) genes in groupⅣ.

A

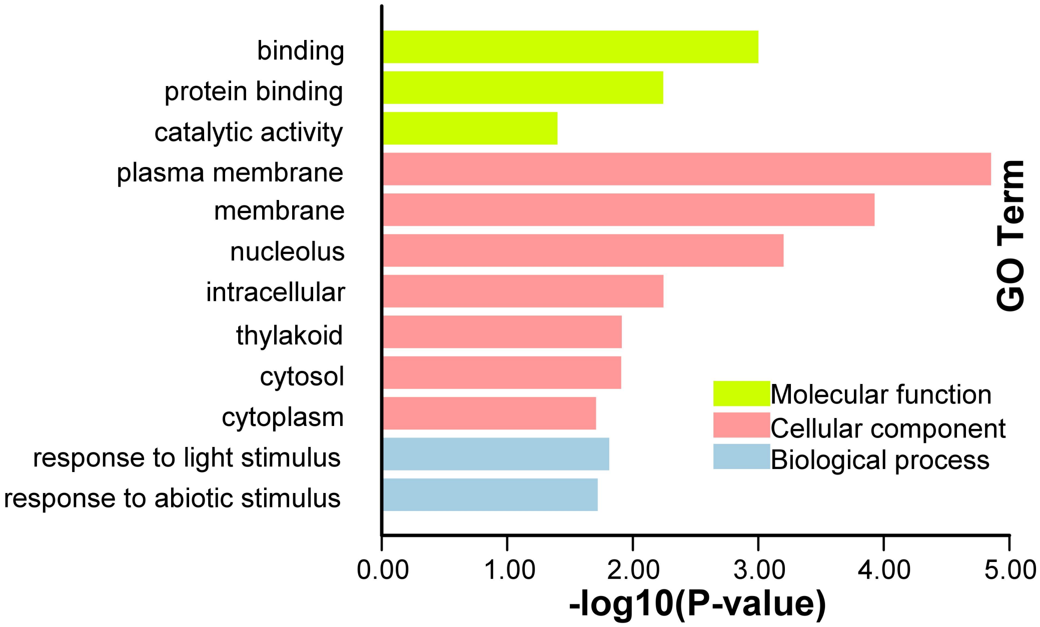

B

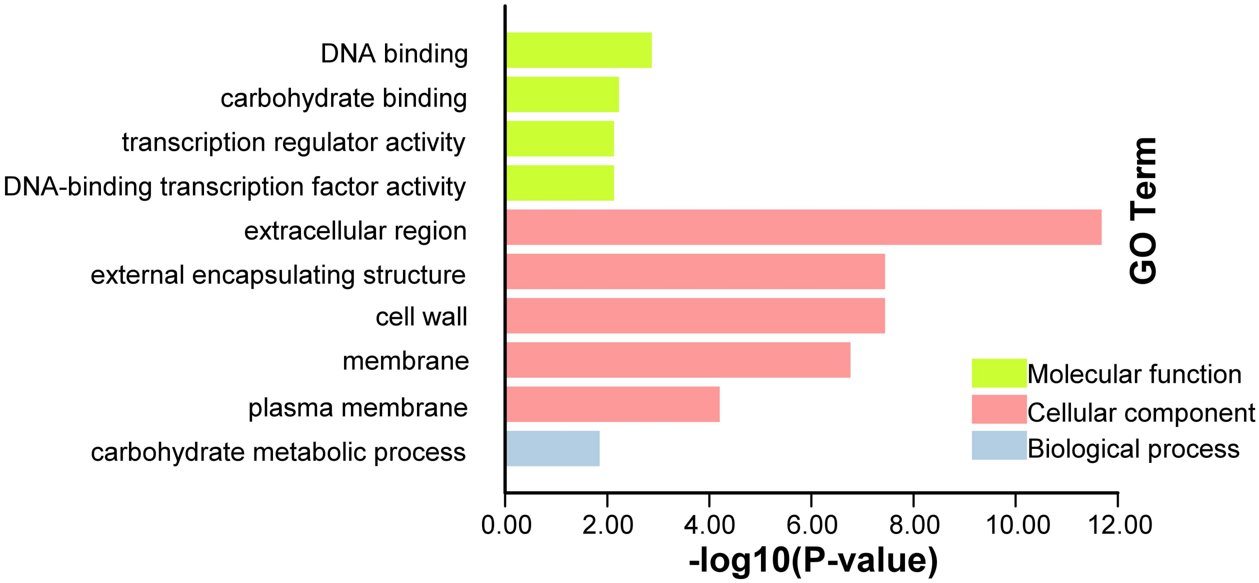

Supplement: Supplementary file 1 — Additional file 1. Fig. S1. Density distribution of raw reads and full-length non-chimeric reads obtained by PacBio Iso-seq. Fig. S2. RT-PCR validation of nine high-confident gene models. Fig. S3. The UpSet plot summarizes the presence of genes in five databases. Fig. S4. The pie diagrams showing the number of transcription factors in different families. Fig. S5. GO enrichment analysis of differentially expressed genes in the comparisons of sporophyll-S1 vs trophophyll-S2, sporophyll-S2 vs trophophyll-S1, and sporophyll-S2 vs trophophyll-S2. Fig. S6. Heatmap showing the differential and non-differential expression of the expansin genes in different pairwise comparisons as obtained from transcriptome analysis. Fig. S7. Phylogenetic tree of expansins in C. chingii and A. thaliana, built with Maximum likelihood. Fig. S8. Analysis of conserved domains of the CcEXP proteins. Fig. S9. Phylogenetic profiling of four groups of expansin genes in 19 species. Fig. S10. Bar plots showing the differentially expressed expansin genes in pteridophyte-specific groups. Fig. S11. GO enrichment analysis of genes that were identified to be significantly coexpressed with CcEXP genes. Fig. S12. Heatmap showing the member number of transcription factors in different families that are coexpressed with at least one CcEXP gene in the four groups. Fig. S13. Yeast two-hybrid assay showing how CcFL01489 interacts with three transcription factor (CcFL09745, CcFL03547, and CcFL06843). Fig. S14. Gene coexpression networks of CrEXP (A) and AtEXP (B) genes of the different groups. Fig. S15. GO enrichment analysis of genes that were identified to be significantly coexpressed with AtEXP (A) and CcEXP (B) genes in groupIV. [file 12915_2023_1743_MOESM1_ESM.pdf]
